# An oomycete NLP cytolysin forms transient small pores in lipid membranes

**DOI:** 10.1126/sciadv.abj9406

**Published:** 2022-03-11

**Authors:** Katja Pirc, Luke A. Clifton, Neval Yilmaz, Andrea Saltalamacchia, Mojca Mally, Tina Snoj, Nada Žnidaršič, Marija Srnko, Jure Borišek, Petteri Parkkila, Isabell Albert, Marjetka Podobnik, Keiji Numata, Thorsten Nürnberger, Tapani Viitala, Jure Derganc, Alessandra Magistrato, Jeremy H. Lakey, Gregor Anderluh

**Affiliations:** 1 Department of Molecular Biology and Nanobiotechnology, National Institute of Chemistry, 1000 Ljubljana, Slovenia.; 2 ISIS Pulsed Neutron and Muon Source, Science and Technology Facilities Council, Rutherford Appleton Laboratory, Harwell Science and Innovation Campus, Didcot, Oxfordshire, Oxford OX11 OQX, UK.; 3 Biomacromolecules Research Team, RIKEN Center for Sustainable Resource Science, Wako, Saitama 351-0198, Japan.; 4 International School for Advanced Studies (SISSA/ISAS), 34136 Trieste, Italy.; 5 Institute of Biophysics, Faculty of Medicine, University of Ljubljana, 1000 Ljubljana, Slovenia.; 6 Department of Biology, Biotechnical Faculty, University of Ljubljana, 1000 Ljubljana, Slovenia.; 7 Theory Department, National Institute of Chemistry, 1000 Ljubljana, Slovenia.; 8 Drug Research Program, Division of Pharmaceutical Biosciences, Faculty of Pharmacy, University of Helsinki, 00014 Helsinki, Finland.; 9 Department of Physics, Chalmers University of Technology, SE-412 96 Gothenburg, Sweden.; 10 Center of Plant Molecular Biology (ZMBP), Eberhard-Karls-University Tübingen, Tübingen, Germany.; 11 Molecular Plant Physiology, FAU Erlangen-Nuremberg, 91058 Erlangen, Germany.; 12 Department of Biochemistry, University of Johannesburg, Auckland Park, Johannesburg, South Africa.; 13 Drug Research Program, Division of Pharmaceutical Chemistry and Technology, Faculty of Pharmacy, University of Helsinki, 00014 Helsinki, Finland.; 14 Chair of Microprocess Engineering and Technology—COMPETE, University of Ljubljana, 1000 Ljubljana, Slovenia.; 15 National Research Council Institute of Material (CNR-IOM), 34136 Trieste, Italy.; 16 Biosciences Institute, Newcastle University, Newcastle upon Tyne NE2 4HH, UK.

## Abstract

Microbial plant pathogens secrete a range of effector proteins that damage host plants and consequently constrain global food production. Necrosis and ethylene-inducing peptide 1–like proteins (NLPs) are produced by numerous phytopathogenic microbes that cause important crop diseases. Many NLPs are cytolytic, causing cell death and tissue necrosis by disrupting the plant plasma membrane. Here, we reveal the unique molecular mechanism underlying the membrane damage induced by the cytotoxic model NLP. This membrane disruption is a multistep process that includes electrostatic-driven, plant-specific lipid recognition, shallow membrane binding, protein aggregation, and transient pore formation. The NLP-induced damage is not caused by membrane reorganization or large-scale defects but by small membrane ruptures. This distinct mechanism of lipid membrane disruption is highly adapted to effectively damage plant cells.

## Teaser

Nep1-like protein from microbial plant pathogen causes membrane disruption by a highly adapted multistep process.

## INTRODUCTION

Plant diseases greatly decrease crop quality and are thus considered one of the most formidable challenges to global food security (*1*, *2*). Microbial plant pathogens secrete a broad spectrum of effector proteins to facilitate infection (*3*, *4*). Necrosis and ethylene-inducing peptide 1 (Nep1)–like proteins (NLPs) constitute a large family of microbial proteins produced by bacteria, fungi, and oomycetes, such as the causal agent of the Great Irish Famine, *Phytophthora infestans* (*5*, *6*). NLP-producing pathogens infect a wide range of important crops, including potato, tomato, soybean, grapevine, and tobacco (*6*).

NLPs are the only known cytolytic pathogen effectors that can permeabilize eudicot plant plasma membranes (*6*–*8*). Recently, major plant membrane constituents, glycosylinositol phosphorylceramides (GIPCs), were identified as the targets of NLP binding to plant plasma membranes (*9*). The molecular mechanism by which NLPs damage plant plasma membranes, however, remains unknown. NLPs are secreted into the apoplast, the interstitial space between plant cells, where ionic strength is generally low, and variations in salt concentration can affect molecular processes at the plant plasma membrane (*10*, *11*). Plant plasma membranes are extremely complex and remain poorly characterized in terms of composition and structure (*12*). Besides GIPCs, which comprise up to 40 mole percent (mol %) of plant lipids and are located in the outer membrane leaflet (*13*), plant membranes also contain substantial amounts of sterols (~30 mol %) (*14*, *15*). GIPCs in the presence of plant sterols were reported to promote formation of lipid phases and be enriched in liquid-ordered domains (*15*).

Known NLPs share a conserved three-dimensional structures (*7*, *16*, *17*). They are single-domain proteins with a central ß sandwich, which is on one side surrounded by three α helices and on the opposite side by three flexible loops. Their negatively charged GIPC-binding cavity contains a bound divalent cation (*7*, *9*). In this study, we used the structurally and functionally well-characterized cytolysin NLP_Pya_ from the phytopathogenic oomycete *Pythium aphanidermatum (**7*, *9**)* to investigate how NLP disrupts lipid membranes.

## RESULTS

### Plant sterols and low-salt concentration promote the binding of NLP_Pya_ to membranes

**Fig. 1.**
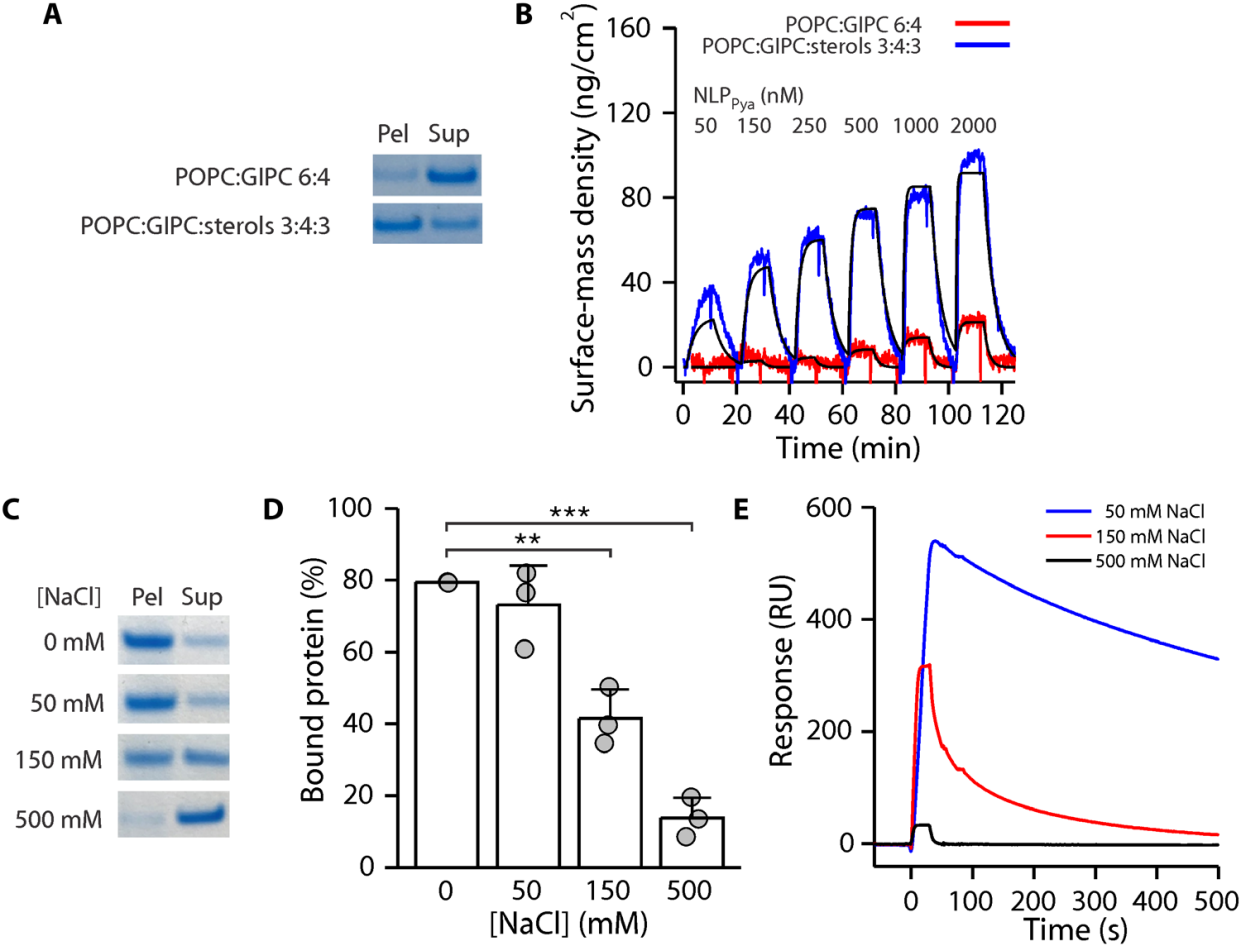
Effect of lipid composition and [NaCl] on NLP_Pya_ association with membranes. (**A**) Binding of NLP_Pya_ to multilamellar vesicles (MLVs) with specific lipid compositions, monitored by sedimentation assay. Pel, pellet; Sup, supernatant. (**B**) QCM kinetic titration experiments. The reported affinities were obtained from a one-site kinetic model fit (black lines). The buffer in (A) and (B) contained 150 mM NaCl. (**C**) Binding of NLP_Pya_ to POPC:GIPC:sterols 3:4:3 MLVs at different [NaCl], monitored by sedimentation assay. (**D**) Quantification of NLP_Pya_ binding from (C). Values are means ± SD (*n* = 3), analyzed with Student’s *t* test (**P ˂ 0.01 and ***P ˂ 0.001). (**E**) Surface plasmon resonance analysis of 250 nM NLP_Pya_ binding to immobilized large unilamellar vesicles (LUVs) at different [NaCl].

We generated model lipid vesicles containing tobacco leaf GIPCs and equimolar mixture of three most abundant plant sterols, β-sitosterol, campesterol, and stigmasterol (*12*, *13*), and first assessed how lipid composition affects the binding of NLP_Pya_. NLP_Pya_ did not bind to 1-palmitoyl-2-oleoyl-*sn*-glycero-3-phosphocholine (POPC) (*9*) or the POPC:sterols bilayer (fig. S1A). However, NLP_Pya_ binding correlated with the increasing amount of GIPCs in the lipid membranes, and the presence of sterols strengthened the interaction (Fig. 1A and fig. S1). We then generated stable supported lipid bilayers (SLBs) (fig. S2) and performed the titration experiment using the quartz crystal microbalance (QCM) assay (Fig. 1B). The presence of sterols substantially increased the affinity of NLP_Pya_, yielding a dissociation constant (*K*_D_) of 162 ± 14 nM for POPC:GIPC:sterols 3:4:3 (molar ratio, all lipid mixtures) compared to 2.3 ± 0.7 μM for POPC:GIPC 6:4 (values are means ± SEM of the fit). The negligible changes in energy dissipation during the titration experiments (fig. S3A) suggest that the interaction is surface-driven, with no measurable restructuring of the membranes even at high (40 μM) protein concentrations (fig. S3B).

Liposome sedimentation assays with increasing [NaCl] further revealed that the interaction of NLP_Pya_ with GIPC-containing liposomes was highly salt dependent and thus predominantly electrostatically driven (Fig. 1, C and D). This was independently confirmed by surface plasmon resonance (Fig. 1E). Furthermore, as opposed to its slow dissociation from GIPC-containing membranes due to specific head group interactions (Fig. 1E), the binding of NLP_Pya_ to control, negatively charged 1-palmitoyl-2-oleoyl-*sn*-glycero-3-phospho-(1′-rac-glycerol) (POPG)–containing bilayers in low salt was easily reversed (fig. S4).

### NLP_Pya_ resides on the membrane surface

**Fig. 2.**
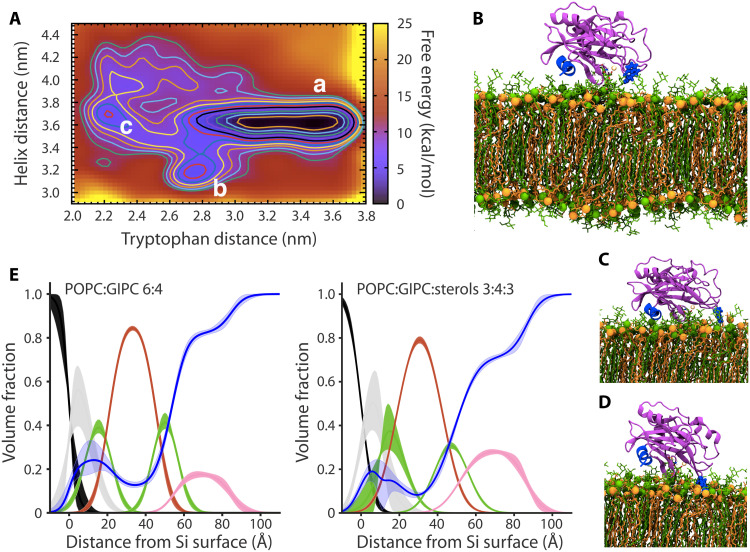
The binding of NLP_Pya_ to membranes. (**A**) Free-energy surface (kcal/mol) of NLP_Pya_ binding to a POPC:GIPC 1:1 membrane, represented as a function of the distance of the tryptophan at position 155 and the distance of the C-terminal helix from the center of mass of the membrane. (**B** to **D**) Three distinct binding poses of NLP_Pya_ on top of the membrane bilayer, corresponding to the free-energy surface minima as labeled in (A) [minima a, b, and c correspond to poses presented in (B), (C), and (D), respectively]. POPC and GIPC lipids are displayed in orange and green, respectively, with the phosphate groups of each shown as van der Waals spheres. The protein is in magenta, the Mg^2+^ is in gold, and the side chain of tryptophan 155 and the C-terminal helix are in blue. Mg^2+^ and tryptophan 155 are presented as van der Waals spheres. (**E**) Component volume fraction profiles determined from the NR data for POPC:GIPC 6:4 and POPC:GIPC:sterols 3:4:3 SLBs after h-NLP_Pya_ binding; substrate layer distributions [silicon (black) and silicon dioxide (gray)], lipid head groups (green), lipid tails (red), protein (magenta), and water distribution across the membrane (blue). Line widths depict the ambiguity in component position and volume fraction as 65% confidence intervals of the acceptable parameter ranges determined from Monte Carlo resampling of the experimental data fits (figs. S7 and S9).

We next explored the binding mode of NLP_Pya_ (Protein Data Bank ID: 3GNZ) to POPC:GIPC 1:1 membranes with all-atom molecular dynamics (MD) simulations. We positioned the GIPC-binding cavity over the GIPC head group and the three loops in contact with the membrane surface, as the loop residue tryptophan at position 155 (W155) is required for cytotoxic activity (*9*). After relaxing this binding pose for 1 μs, we performed enhanced sampling metadynamics simulations to accelerate the exploration of other possible NLP_Pya_ binding poses and estimate their relative free energies (*18*). After 1 μs of simulation, NLP_Pya_ adopted three distinct binding modes, all positioning the protein on the membrane surface and exhibiting the carboxylic acid of GIPC’s glucuronic acid moiety stably bound with the Mg^2+^-ion placed within the NLP_Pya_ cavity (fig. S5). In the most stable pose, the C-terminal helix of NLP_Pya_ lies on top of the membrane (minimum a in Fig. 2, A and B), whereas in the other two metastable minima, b and c (Fig. 2A), the C-terminal helix more deeply penetrates into the GIPC head group region (Fig. 2C), or W155 more closely interacts with the GIPC head groups (Fig. 2D), respectively. The simulations strikingly reveal a rough free-energy surface underlying the binding of NLP_Pya_ to the membrane model, in which the free energy required to leave the main minimum is 7.0 ± 0.6 kcal/mol and all metastable states lie within 5.0 ± 0.6 kcal/mol above it. Consistent with the [NaCl] dependence of the NLP_Pya_-GIPC interaction (Fig. 1, C to E), a calculation of the interaction energies between the membrane and NLP_Pya_ reveals that the electrostatic interactions mostly contribute to stabilizing the protein-binding pose, although hydrophobic interactions also form (fig. S5 and table S1). Several persistent hydrogen bonds are established between the GIPC head groups and NLP_Pya_ residues from the loops surrounding the binding cavity and from the C-terminal helix (fig. S5 and table S2). Thus, our MD simulations suggest how NLP_Pya_ binds strongly to GIPC lipids and predict partial insertion into the outer head group region.

We used neutron reflectometry (NR) to experimentally test the MD simulations, as this approach can yield detailed penetration profiles for membrane-bound proteins (*19*). Membrane interactions were examined independently with natural hydrogenous (h-NLP_Pya_) and deuterated (d-NLP_Pya_) proteins (fig. S6). d-NLP_Pya_ was used to resolve the protein within the hydrogen-rich, hydrophobic, membrane core of SLBs. For the POPC:GIPC 6:4 bilayer, NR revealed that a ~29-Å-thick layer of h-NLP_Pya_, corresponding to the thickness of a monomer, formed on the outer membrane surface (Fig. 2E, fig. S7, and table S3). Consistent with the MD simulations (Fig. 2, B to D, and fig. S5), the protein was not detected within the membrane core, with only a minor component in the outer bilayer head group region (Fig. 2E and table S3). The interaction of d-NLP_Pya_ with the bilayer was similar to that of h-NLP_Pya_, creating a ~26-Å-thick layer on the membrane surface (fig. S8A and table S3). The interaction of h-NLP_Pya_ and d-NLP_Pya_ with the POPC:GIPC:sterols 3:4:3 bilayer was qualitatively similar; the protein was only detected on the surface of the membrane and not in the membrane core (Fig. 2E, figs. S8B and S9, and table S3), whereas no h-NLP_Pya_ was identified on the POPC:sterols membrane (fig. S10 and table S3).

### NLP_Pya_ associates into assemblies that display shallow membrane penetration

**Fig. 3.**
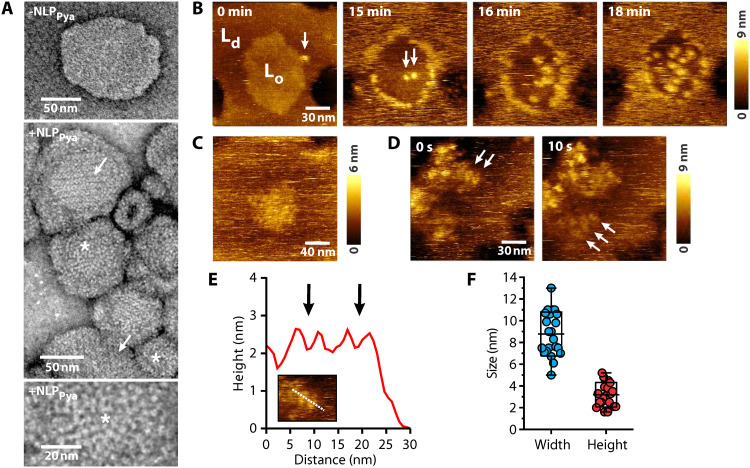
Imaging of NLP_Pya_ bound to the membrane surface. (**A**) TEM images of 500 nM NLP_Pya_ bound to liposomes composed of POPC:GIPC:sterols 1:6:3 reveal particles and circular aggregates (*) and ordered assemblies (arrow). (**B**) HS-AFM images of the DOPC:GIPC:sterols 1:1:1 SLB, consisting of L_o_ and L_d_ phases, in the presence of 300 nM NLP_Pya_. Arrows indicate the protein clusters at the phase boundary of the membrane at 0 min, when protein was added, and on the L_o_ domain at 15 min. (**C**) NLP_Pya_ clusters on an L_o_ domain. (**D**) NLP_Pya_ clusters imaged at two different time frames. (**E**) The height profile is for the dashed line in the inset and reveals individual circular aggregates (arrows). (**F**) Quantification of cluster diameter and height (*n* = 22). The boxes show means ± SD, and whiskers show minimal and maximal values.

We imaged membrane-associated NLP_Pya_ with transmission electron microscopy (TEM) and high-speed atomic force microscopy (HS-AFM). TEM of negatively stained samples revealed GIPC-containing liposomes covered with protein in the form of particles and circular aggregates, which were sometimes arranged in ordered assemblies (Fig. 3A). HS-AFM imaging monitored the binding and assembly of NLP_Pya_ on 1,2-dioleoyl-*sn*-glycero-3-phosphocholine (DOPC):GIPC:sterols and POPC:GIPC:sterols SLBs with phases that separated into liquid-ordered (L_o_) and liquid-disordered (L_d_) domains (Fig. 3B and figs. S11 and S12). NLP_Pya_ preferentially associated with GIPC in the L_d_ phase, then accumulated at the phase boundary, and last formed tightly packed clusters on the GIPC-rich L_o_ domains (Fig. 3, B and C; figs. S11 and S12; and movies S1 to S3), similar to the ordered assemblies observed with TEM (Fig. 3A). Clusters of 9 ± 2 nm in diameter and 3 ± 1 nm in height, with a central depression, were observed on the membranes (Fig. 3, D to F). The thickness of the protein layer on the membrane thus agrees with the MD simulations and NR data (Fig. 2, A to E, and table S3). The large standard deviation, especially in cluster width (Fig. 3F), might be due to the variation in cluster stability and mobility, as assembled proteins were easily deformed or displaced by the HS-AFM probe because of their shallow binding to the membrane (Fig. 3D and fig. S13). During the binding and assembly of NLP_Pya_, the GIPC-rich L_o_ domains neither merged nor reorganized, even after full coverage with NLP_Pya_ (figs. S11 and S12 and movies S1 and S3).

### NLP_Pya_ induces formation of transient pores permeable for small molecules

**Fig. 4.**
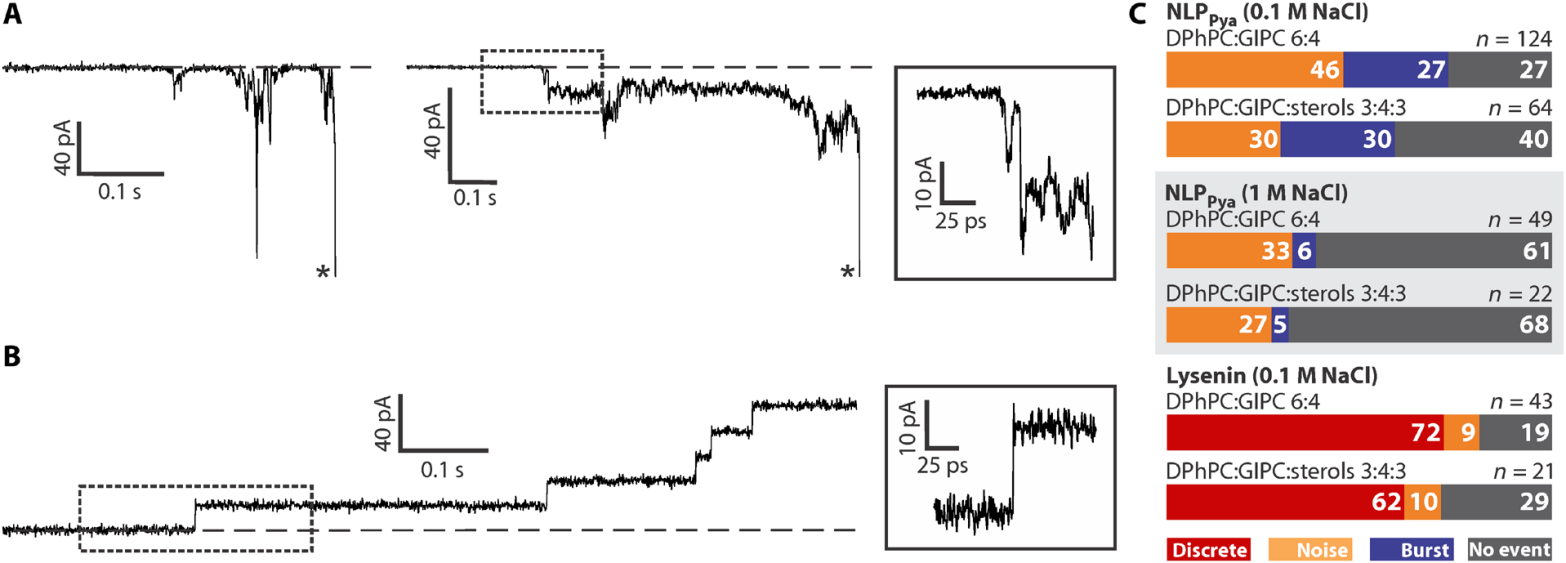
NLP_Pya_ pore formation in planar lipid bilayers. (**A**) Two typical current traces (qualified as noise) observed by current recording experiments when planar lipid bilayers composed of 1,2-diphytanoyl-*sn*-glycero-3-phosphocholine (DPhPC):GIPC 6:4 were exposed to 9.4 μM NLP_Pya_. The applied potential was −100 mV. The asterisks denote membrane rupture. The dash-framed part of the trace is enlarged on the right. (**B**) Typical current trace when planar lipid bilayers were exposed to 41.4 pM lysenin pores. The membrane composition was the same as in (A). The applied potential was +50 mV. The dash-framed part of the trace is enlarged on the right. (**C**) Quantification of the experiments shown in (A) and (B). The numbers in the columns denote the percentages of each observed event.

We then used a planar lipid bilayer approach and measurements of ionic currents (*20*) to assess the NLP_Pya_-induced damage of the membrane. NLP_Pya_-induced membrane openings (Fig. 4A) differ from discrete membrane openings induced by typical pore-forming toxins such as lysenin (Fig. 4B), which forms well-defined β-barrel pores of ~2 nm in diameter (*21*). While the presence of sterols in the membrane increased the affinity of NLP_Pya_ (Fig. 1B), it did not alter its membrane-disrupting capacity (Fig. 4C). NLP_Pya_-induced permeability was less frequent at high [NaCl] (Fig. 4C), in agreement with the binding studies (Fig. 1, C to E).

**Fig. 5.**
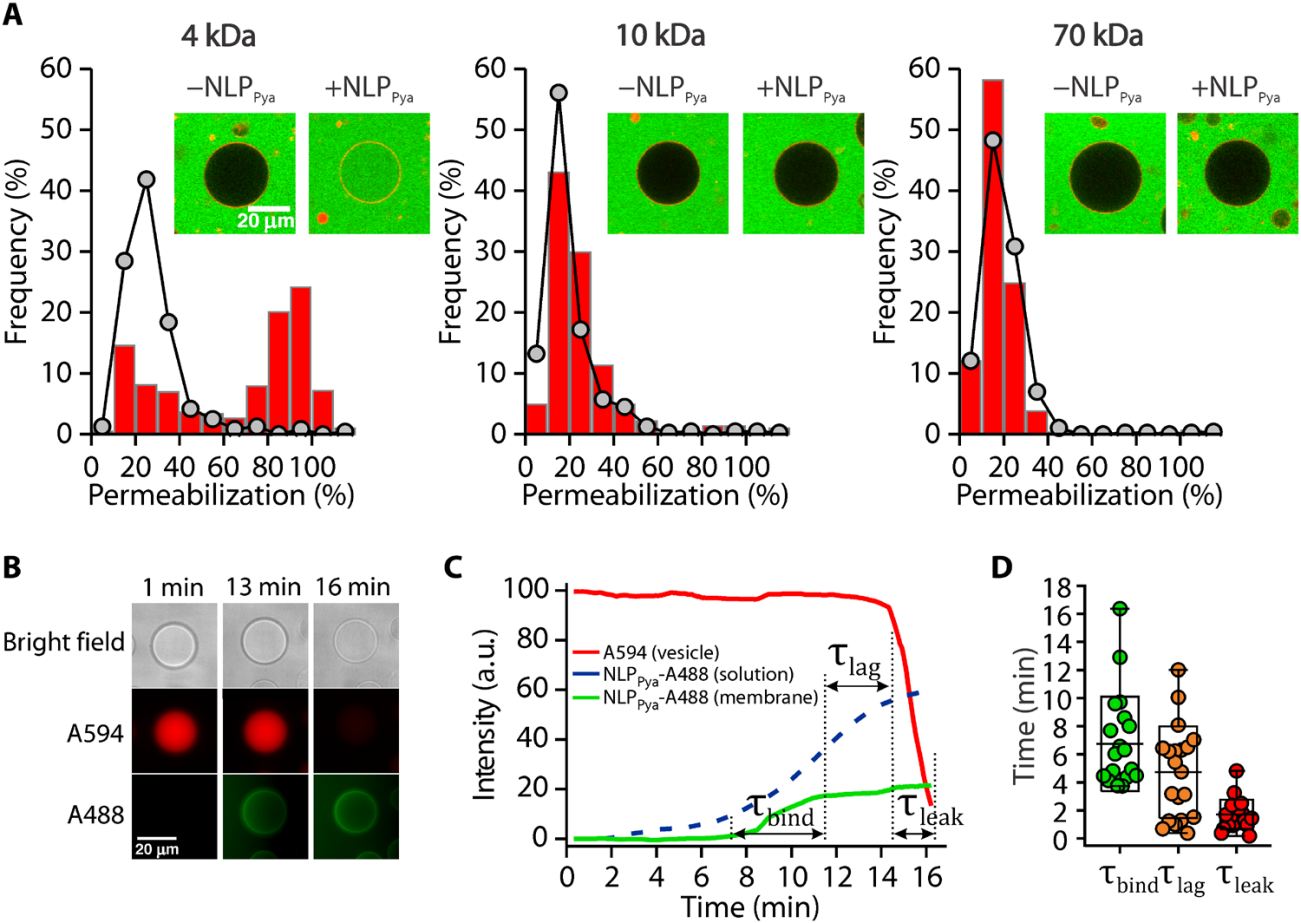
NLP_Pya_-induced permeabilization of GUVs. (**A**) GUVs (POPC:GIPC 1:1.2) imaged in the presence (red columns) and absence (gray dots) of 500 nM NLP_Pya_ and different-sized FDs. For each condition, a representative GUV is shown. The number of GUVs analyzed was 239 to 633. (**B** to **D**) Real-time monitoring of NLP_Pya_ binding and membrane permeabilization of POPC:GIPC 1:1.75 GUVs in a microfluidic diffusion chamber. (B) Time-lapse images of a representative vesicle filled with A594 (middle row, red channel) and exposed to an increasing concentration of NLP_Pya_-A488 (bottom row, green channel). (C) Time dependence of the fluorescent signals in (B). (D) Quantification of the binding times (τ_bind_), time lags (τ_lag_), and leak times (τ_leak_) for the microfluidic experiments presented in (C). Data for individual vesicles are presented with dots (*n* = 21). The boxes show means ± SD, and whiskers show minimal and maximal values. a.u., arbitrary units.

To estimate the size of the NLP_Pya_ pore, we imaged giant unilamellar vesicles (GUVs) along with differently sized fluorescent dextrans (FDs). NLP_Pya_ induced pores permeable to 4-kDa FD (~2.8 nm in diameter), but not 10- and 70-kDa dextrans (4.6 and 12 nm in diameter, respectively) (Fig. 5A). NLP_Pya_ membrane attachment did not affect GUV morphology, as the vesicles neither disintegrated nor collapsed during the time frame of the experiments.

Last, the binding of Alexa Fluor 488–labeled NLP_Pya_ (NLP_Pya_-A488) to GIPC-containing GUVs and the subsequent membrane permeabilization were observed in real time in a custom-developed microfluidic diffusion chamber (fig. S14A) (*22*). GUVs in low [NaCl] filled with Alexa Fluor 594 (A594) were monitored with time-lapse microscopy using two fluorescence channels (Fig. 5B and movie S4). The NLP_Pya_-A488 signal on the membrane was coupled to the increasing concentration of NLP_Pya_-A488, diffusing into the chamber, and exhibited a sigmoidal shape with a binding time (τ_bind_) of 6.7 ± 3.3 min (Fig. 5, C and D, and fig. S14B). Several minutes later, the A594 signal in the vesicles decreased, indicating the leakage of A594 molecules out of the vesicle due to membrane permeabilization. This time lag (τ_lag_) between the saturation of the NLP_Pya_-A488 signal on the membrane and the onset of membrane permeabilization was 4.4 ± 3.4 min (Fig. 5, C and D, and fig. S14B). The leakage (τ_leak_) of A594 out of the vesicle was a relatively fast process (1.7 ± 1 min). When the NLP_Pya_-A488 solution was washed from the chamber, the signal on the membranes remained unchanged (fig. S14C). No binding of the protein was observed on pure POPC GUVs (fig. S14D).

## DISCUSSION

Here, we provide insight into the unique molecular mode of action of membrane damage for a member of a large family of plant pathogen virulence effectors (*7*, *8*). On the basis of our results, we suggest that NLP_Pya_-induced membrane damage occurs in several successive steps (fig. S16) composed of a predominantly electrostatically driven, initial association with membrane lipid receptors, GIPCs (Figs. 1 and 2), followed by protein clustering on the membrane plane (Fig. 3) and formation of transient heterogeneous pores that are permeable to small molecules (Figs. 4 and 5). Sterols may affect the availability of the GIPC head groups for NLP_Pya_ binding, similar to cholesterol that modulates conformation of glycosphingolipid head groups (*23*), and allow the formation of tightly clustered NLP_Pya_ aggregates on L_o_ domains (Fig. 3).

An important advantage of the real-time observations and the analysis of the experiments in the microfluidic system is that they allow for the testing of different kinetic models of the mechanisms of membrane permeabilization (text S1 and fig. S15). In the simplest model, it can be assumed that membrane permeability is proportional to the concentration of membrane-bound NLP_Pya_. Modeling revealed that regardless of the value of the permeability constant, it is not possible to reproduce the observed time lag between the NLP_Pya_ saturation on the membrane and the onset of the solute leakage out of the vesicles within this model (fig. S15A). The observed time lag hints that membrane permeabilization is linked to a stochastic process on the membrane. For example, a stochastic formation of a large membrane rupture could lead to the observed evolution of the fluorescent signals in Fig. 5C. However, such a membrane rupture would also lead to a leakage of large molecules, which is not consistent with our observations (Fig. 5A). This sigmoidal kinetic process, with a time lag, is reminiscent of those observed in various processes of protein aggregation, such as formation of functional oligomers of pore-forming toxins (*24*) or the growth of amyloid-β or α-synuclein aggregates related to neurodegenerative diseases (*25*). According to a minimal two-step model that was proposed for modeling these phenomena (*26*), membrane-bound NLP_Pya_ undergoes a slow nucleation process that then triggers a fast protein clustering. Assuming that membrane permeability is proportional to the amount of clustered proteins (as if, for example, each protein cluster comprised a fixed density of small pores that allow the leakage of small molecules), we were readily able to reproduce the observed behavior (fig. S15B). The observed time lag between the NLP_Pya_ saturation on the membrane and the membrane permeabilization (Fig. 5, C and D) thus indicates that there is a step that involves association of NLP_Pya_ at the surface of the membrane and agrees with TEM, HS-AFM, and the measurements of electrical conductivities (Figs. 3 and 4).

While functionally reminiscent of bacterial membrane damage induced by cationic peptides via a carpet mechanism (*27*), NLP_Pya_ membrane damage differs because of its lipid specificity. β-barrel pore-forming proteins often form prepore intermediates that are distinct in height from the inserted final pores (*21*, *28*). Conversely, no significant changes in the thickness of the protein layer on the membrane were observed (Figs. 2 and 3), implying that NLP_Pya_ does not undergo substantial conformational changes or deeper insertion into the membrane to induce membrane damage. The current noise observed during our planar bilayer measurements indicates that NLP_Pya_-induced membrane damage is in the form of transient ruptures that are permeable to small molecules (Figs. 4 and 5). The NLP_Pya_-induced permeabilization may be formed via shallow bilayer penetration and local GIPC reorganization due to numerous contacts between NLP_Pya_ and GIPC molecules (figs. S5 and S16 and table S2). According to the MD simulations, the putative regions of NLP_Pya_ that are responsible for formation of pores are the loops around the GIPC-binding site and the C-terminal helix (Fig. 2, B to D, and fig. S5). NLP_Pya_ thus exerts a unique molecular mechanism for membrane damage that is different from that of structurally related actinoporins (*7*) or unrelated cytolysins of bacterial origin (*Bt*-toxins, anthrax toxin, *Staphylococcus aureus* alpha toxin family, colicin family, aerolysin-like toxins, HlyA and similar toxins, etc.) (*21*, *28*–*30*), which form transmembrane pores by clusters of helices or beta barrels (*30*).

Membrane damage via the formation of transmembrane pores is commonly used to damage animal cells (*29*–*33*) but rarely observed for plant pathogens. NLP_Pya_-induced membrane disruption must thus be highly adapted to function in a plant membrane environment characterized by highly negatively charged plant plasma membranes (*12*), a specific lipid receptor (GIPC) with adjacent sterols (*9*), and low apoplastic ionic strength (*11*). The relatively small size of the membrane ruptures indicates that the cytotoxic activity of NLPs may provide pathogens with ions and small molecular weight nutrients derived from damaged plant cells, thus enhancing virulence of necrotrophic pathogens. Insights into the molecular mechanisms underlying NLP-induced pore formation will unlock new avenues for developing specific and targeted strategies to inhibit NLP and thus for combating NLP-producing microbial pathogens (*34*).

## MATERIALS AND METHODS

### Lipid material

The lipids POPC, POPG, DOPC, DPhPC, β-sitosterol, and stigmasterol were supplied from Avanti Polar Lipids (Alabaster, AL, USA). β-Sitosterol for HS-AFM experiments and campesterol were purchased from Sigma-Aldrich Inc. (St. Louis, MO, USA). Rhodamine-labeled 1,2-dihexadecanoyl-*sn*-glycero-3-phosphoethanolamine (rhodamine-DHPE) was purchased from Thermo Fisher Scientific (USA).

### Expression and purification of NLP_Pya_

The NLP_Pya_ protein was expressed and purified as described previously (*7*, *9*, *35*). The production of deuterated d-NLP_Pya_ for NR experiments followed the same protocol, with the following modifications. The initial adaptation of *Escherichia coli* BL21 (DE3) to D_2_O (Cambridge Isotopes Lab Inc.) was achieved with four sequential precultures over 3 days with increasing D_2_O levels in M9 medium supplemented with 1 mM MgSO_4_, 0.1 mM CaCl_2_, 0.1 mM MnCl_2_, 50 μM ZnSO_4_, 50 μM FeCl_3_, vitamins (pyridoxine, biotin, d-panthotenic acid, folic acid, choline, niacinamide, riboflavin, and thiamine), ampicillin (100 μg/ml) as a selection marker, and 0.2% (w/v) glucose (nondeuterated) as the sole carbon source. LB H_2_O medium was inoculated with clones from a fresh transformation plate (*9*) and incubated at 37°C and 200 rpm (preculture 1). In the evening, the supplemented M9 H_2_O medium was inoculated with preculture 1 and incubated overnight (preculture 2). The next day, supplemented M9 50% D_2_O was inoculated with preculture 2 (preculture 3) and used in the evening to inoculate supplemented M9 D_2_O (preculture 4). The next morning, preculture 4 was used to inoculate ~1.8 liters of M9 D_2_O. When the culture reached OD_600_ (optical density at 600 nm) of ~0.8, 0.5 mM isopropyl-β-d-thiogalactopyranoside was added to induce protein production. Cells were cultivated at 26°C and 180 rpm for 15 hours. The protein was purified with ion-exchange chromatography on a gravity flow nickel affinity column (Ni-NTA Superflow, Qiagen) (*35*). Protein-containing fractions were pooled and dialyzed against D_2_O.

The purity of the nondeuterated (h-NLP_Pya_) and deuterated (d-NLP_Pya_) protein preparations was verified by SDS–polyacrylamide gel electrophoresis (SDS-PAGE; fig. S6A). The activities of the proteins were identical, as measured by infiltration assay (fig. S6B). The proteins also had comparable circular dichroism and temperature melting profiles (fig. S6, C and D) and bound to GIPC-containing vesicles in a similar manner (fig. S6E).

### Preparation of GIPCs

GIPCs were extracted and purified as previously described (*9*, *36*). Briefly, tobacco (*Nicotiana tabacum*) leaves were blended with cold 0.1 N aqueous acetic acid and filtered through miracloth. The slurry was extracted with hot 70% ethanol/0.1 N HCl. The pellet was washed with cold acetone and diethyl ether to yield a whitish precipitate, which was then dissolved in tetrahydrofuran:methanol:water (4:4:1, v:v:v) containing 0.1% formic acid. Dried precipitate was submitted to a butan-1-ol:water (1:1, v:v) phase partition. Residue from the upper GIPC-containing butanol phase was dissolved in tetrahydrofuran:methanol:water (4:4:1, v:v:v) containing 0.1% formic acid. GIPCs were characterized by matrix-assisted laser desorption/ionization mass spectrometry (*36*), and their mass was estimated from their dry weight. To test 40 μM NLP_Pya_ in the QCM experiment, SLB was prepared from tomato GIPCs, purified in the same way as described above.

### Liposome preparation

Liposomes were prepared by mixing POPC or DOPC with tobacco GIPCs and/or an equimolar mixture of the sterols β-sitosterol, campesterol, and stigmasterol. The final concentration of sterols in the lipid mixture was kept constant at 30 mol % (*15*). For some liposome sedimentation and surface plasmon resonance experiments, lipid mixtures of POPC and POPG were used. Multilamellar vesicles (MLVs) were prepared by hydration of thin lipid films in appropriate warm buffer and vortexing of the lipid suspensions in the presence of glass beads. Small unilamellar vesicles (SUVs) were obtained by sonication of MLVs on ice for a total of 30 min, with 10-s on-off cycles at 40% amplitude using Vibracell Ultrasonic Disintegrator VCX 750 (Sonics and Materials, Newtown, USA). Large unilamellar vesicles (LUVs) were prepared from MLVs by several freeze/thaw cycles and subsequent extrusion through polycarbonate filters with 50- or 100-nm pore size using a two-syringe extruder (NanoSizer Extruder, T&T Scientific, USA). The vesicle size distribution was routinely checked by dynamic light scattering (Zetasizer Nano-ZS, Malvern Instruments, UK).

### Liposome sedimentation assay

Liposome sedimentation assay was performed as previously described (*9*, *17*), with some modifications. Briefly, NLP_Pya_ (0.0625 mg/ml) was incubated with MLVs (5 mM) composed of either POPC, GIPCs, and/or sterols or of POPC and POPG for 30 min and 600 rpm at room temperature. The effects of sterols and salt on protein binding were tested in 20 mM MES and 150 mM NaCl (pH 5.8) and in 20 mM MES and 2 mM EDTA (pH 5.8) containing 0, 50, 150, or 500 mM NaCl. The mixture was centrifuged, and the liposomes were washed twice with the same buffer and subjected to SDS-PAGE, followed by Coomassie Brilliant Blue staining. Protein bands were statistically analyzed after densitometric quantification using an image analyzer system [ImageJ 1.29×, National Institutes of Health (NIH), USA]. Each value represents the average of three independent experiments.

### QCM measurements

SUVs (2 mg/ml) were diluted to 0.15 mg/ml in buffer [20 mM MES, 150 mM NaCl (pH 5.8), and 5 mM CaCl_2_], which was added to promote the rupture of vesicles to form SLBs (*37*). The impedance-based QCM Z-500 instrument (KSV Instruments Ltd., Helsinki, Finland) was used with its own peristaltic pump system (Ismatec/Cole-Parker GmbH, Wertheim, Germany). Vesicles were flown over the QCM crystals (Q-Sense Inc./BiolinScientific, Västra Frölunda, Sweden) for 5 to 10 min at flow speeds of 250 μl/min. After 5-min injections of buffer and buffer with 5 mM EDTA, two consecutive injections of ultrapure water were performed to induce osmotic shock to rupture the remaining vesicles on the surface. After reducing the flow speed to 75 μl/min, increasing concentrations (50, 150, 250, 500, 1000, and 2000 nM) of NLP_Pya_ were sequentially injected for 10 min. Each injection was followed by a 10-min dissociation phase. Sensors were cleaned in situ with sequential injections of 20 mM CHAPS, 2% (v/v) Hellmanex, 50% (v/v) ethanol, and ultrapure water.

Data were analyzed with in-house Python scripts using the rigid (Sauerbrey) layer model. When required, baseline corrections for the data were performed using OriginPro software (v. 2018b, OriginLab Corp., Northampton, MA, USA), and the data were interpolated to set a common time axis. Kinetic titration data corresponding to the hydrated surface mass density were fitted using the one-site kinetic model in TraceDrawer software (v. 1.9.1, Ridgeview Instruments AB, Vänge, Sweden).

### Surface plasmon resonance

The binding of NLP_Pya_ to POPC:GIPC:sterols 3:4:3 (n:n:n) lipid bilayers was monitored with Biacore X100 (GE Healthcare) and the sensor chip L1 (GE Healthcare) (*38*, *39*). The binding was tested at three different salt concentrations using running buffer [20 mM MES and 2 mM EDTA (pH 5.8), with 50, 150, or 500 mM NaCl]. The system was first primed with the running buffer. After regenerating the chip, POPC:sterols 7:3 (n:n) LUVs (0.1 mg/ml diluted in running buffer) were injected over the first flow cell, while POPGIPC:sterols 3:4:3 (n:n:n) LUVs (0.1 mg/ml diluted in running buffer) were captured on the second flow cell. Both lipid vesicles were loaded at 5 μl/min to 3000 response units (RU). The surface was then conditioned with 60- and 30-s injections of bovine serum albumin (0.1 mg/ml) at 10 μl/min. The protein (250 nM) was injected manually at 10 μl/min for 30 s, followed by a 10-min dissociation phase. The binding of NLP_Pya_ to POPC:POPG 6:4 (n:n) lipid bilayers was conducted in the same manner as described above, with POPC LUVs captured on the first flow cell and POPC:POPG 6:4 (n:n) LUVs captured on the second flow cell.

### Circular dichroism spectroscopy

Far-ultraviolet circular dichroism spectra of h-NLP_Pya_ and d-NLP_Pya_ (0.25 mg/ml) were recorded using a 0.1-cm path length quartz cuvette on a Chirascan spectrometer (Applied Photophysics) in ultrapure water and D_2_O, respectively. Spectra were measured in a 200- to 250-nm wavelength range with a step size of 0.5 nm and an integration time of 1 s at 20°C. Each spectrum represents the average of 10 scans. Spectra were baseline-corrected, smoothed, and processed with the Chirascan software. Spectral units were expressed as the mean molar ellipticity per residue.

### Thermal stability assay

The thermal stability of h-NLP_Pya_ and d-NLP_Pya_ (0.1 mg/ml) was measured with the differential scanning fluorimetry (Thermofluor) assay using 10x SYPRO Orange dye (Thermo Fisher Scientific). The thermal stability of proteins was tested in 20 mM MES (pH 5.8) prepared in ultrapure water (for h-NLP_Pya_) or D_2_O (for d-NLP_Pya_). Samples were subjected to a temperature gradient from 25°C to 95°C at steps of 1°C/min. Temperature melting profiles were acquired with LightCycler 480 (Roche). Melting temperatures (*T*_m_) were determined using the Boltzmann function in Origin 8.1 (OriginLab). Each *T*_m_ value represents the average of six repeats in three independent experiments.

### Infiltration assay

The infiltration assay was performed on tobacco plants (*N. tabacum* “White Burley”). h-NLP_Pya_ and d-NLP_Pya_ (50 μl of 500 nM) in ultrapure water and D_2_O, respectively, were infiltrated abaxially into tobacco leaves using a syringe without a needle. Lesion formations were examined 24 hours after infiltration.

### MD simulations

Starting from the experimentally determined structure of NLP_Pya_ with a Mg^2+^ ion trapped in the central cavity (Protein Data Bank ID: 3GNZ) (*7*), we manually docked the protein onto the GIPC sugar head group (*9*) in the center of a POPC:GIPC 1:1 membrane bilayer so that the sugar head group (i.e., the glucuronic acid and glucosamine moieties) was inside the Mg^2+^-binding cavity. The protein was initially placed on the membrane so that its tryptophane at position 155 (W155), which was demonstrated to be important for the cytolytic activity of NLP_Pya_ (*9*), was near a preequilibrated membrane surface. The membrane was built by the membrane generator MemGen (*40*). The symmetric membrane bilayer contained 80 GIPC molecules and 80 POPC molecules. The system was solvated by a layer of 30 Å of water molecules in the *z* direction, leading to a total of 61,461 atoms.

The system topology was built with the tleap module of the amber tools 18 using the Amber parm99SB parameters for the protein (*41*) and lipid17 parameters for POPC. GIPC was parametrized according to the following procedure: The GIPC molecule was geometry-optimized with the density functional theory with the B3LYP exchange correlation function and the 6-31G* basis set using the Gaussian09 program (*42*). Charges were calculated according to the Restrained Electrostatic Potential (RESP) approach with the antechamber module of Amber18 tools (*43*). The other parameters were determined with the antechamber tool of Amber18 using the general amber force field parameters for organic molecules (*44*). The protonation states of the residues were determined with the Propka web server (*45*). The water molecules were described using the TIP3P water model (*46*). Charge neutrality was achieved by the addition of 76 Na^+^ ions described with the Joung and Cheatman parameters (*47*). The Mg^2+^ ion in the binding cavity was described with the Allner *et al.* parameters (*48*). The system topology was then converted to the GROMACS format with the acpype software (*49*).

Preliminary energy minimization was done with the steepest descent algorithm. An initial equilibration of the membrane was performed for 100 ns, keeping the protein atoms harmonically restrained with a force constant of 1000 kJ/mol nm^2^, until a constant value (92 Å × 92 Å × 151 Å) of the simulation box size was reached. Constraints were then released, and the system was slowly thermalized to the target temperature of 300 K during a 10-ns MD. Last, the system was further equilibrated for 1 μs.

For all MD simulations, the pressure was kept to equilibrium value with the Parrinello-Rahman barostat (*50*), while the temperature was controlled with the velocity rescale thermostat (*51*). MD simulations were performed using GROMACS 2018.2 (*52*) and an integration time step of 2 fs. All covalent bonds involving hydrogen atoms constrained with the LINCS algorithm (*53*). The particle mesh Ewald scheme (*54*) was used to account for electrostatic interactions.

Well-tempered metadynamics simulations (*55*) were performed on the equilibrated trajectory using the plumed 2.4.3 plugin (*56*). In these simulations, two collective variables (CVs) were used, describing the distance of the tryptophan at position 155 (W155) and C-terminal helix centers of masses to the center of mass of the membrane (CV1 and CV2, respectively) to account for their penetration into the membrane bilayer. CV1 was defined as the *z* projection of the distance of the center of mass of W155 to the center of mass of the membrane, and CV2 was defined as the distance of the center of mass of the C-terminal helix of NLP_Pya_ (residues 200 to 210) to the center of mass of the membrane. In the simulations, we used Gaussian hills of heights of 1.2 kJ/mol and sigma of 0.08 Å and 0.1 Å for CV1 and CV2, respectively. The bias factor was 30, and the Gaussian deposition rate was one every 500 steps.

The error of the free-energy profile was calculated as the SD of different time averages of CVs from different simulation blocks. The number of blocks was chosen using the block analysis technique to obtain uncorrelated data, i.e., looking at the average error as a function of the block sizes until reaching a plateau.

Interaction energies were calculated with the g_energy tool, while the root mean square deviation was monitored with the gmx_rms tool of GROMACS 2018.2. The hydrogen (H)–bonds were calculated with AMBER’s *cpptraj* tool. The fatslim program (*57*) was used to calculate the tail thickness and area per lipid of the membrane: 31.8 ± 0.28 Å and 59.6 ± 0.4 Å^2^, respectively. This is in good agreement with the measured values reported in table S3.

### Neutron reflectometry

Neutron reflectometry (NR) measurements were performed with the white beam INTER reflectometer (*58*) at the ISIS Spallation Neutron Source, Rutherford Appleton Laboratory (Oxfordshire, UK), which uses neutron wavelengths of 1 to 16 Å. The reflected intensity was measured at glancing angles of 0.7° and 2.3°. Reflectivity was measured as a function of the momentum transfer: *Q*_z_ = (4π sin θ)/λ, where λ is the wavelength and θ is the incidence angle. Data were obtained at a nominal resolution (*dQ*/*Q*) of 3.5%. The total illuminated sample length was ~60 mm. Liquid flow and liquid chromatography systems were set up as previously described (*19*). Briefly, solid liquid flow cells were placed onto the instrument sample position and connected to instrument-controlled high-performance liquid chromatography (HPLC) pumps (Kauer Smartline 1000), which controlled changes in the solution, i.e., isotopic contrast as well as counter-ion concentration and type in the flow cells.

During the NR measurements, the membrane-free, clean, silicon surfaces were initially measured in 20 mM MES (pH 5.8) D_2_O and 20 mM MES (pH 5.8) H_2_O (hereafter D_2_O buffer and H_2_O buffer, respectively). The solid liquid flow cells were then heated to 35°C, and freshly sonicated SUVs (0.1 mg/ml) in 2 mM CaCl_2_ D_2_O solution were injected into the flow cells. The formation of SLBs was monitored by NR. Upon confirmation of SLB formation, the cells were cooled back to 20°C and flushed with D_2_O buffer to remove excess vesicles. The bilayers were then examined by NR in 100% D_2_O, 80% D_2_O:20% H_2_O, silicon-matched water (Si-MW; 38% D_2_O:62% H_2_O), and 100% H_2_O buffer solutions. NLP_Pya_ (500 nM) in H_2_O buffer was then flushed into the solid liquid flow cell. The NR data in this contrast were used to assess the binding of both d-NLP_Pya_ and h-NLP_Pya_ to the membrane. Then, data were collected for the same set of contrasts for the proteo-membrane complex and for the membrane alone.

### NR data analysis

NR data were analyzed using the RasCal fitting package (version 2014b, A. Hughes, ISIS Spallation Neutron Source, Rutherford Appleton Laboratory), which uses an optical matrix formalism (*59*, *60*) to fit layer models of the interfacial structure to the experimental reflectivity data. The program is designed to support the simultaneous fitting of multiple sets for which parameters, such as the layer thickness and roughness (grading of either the bulk interface or interface between layers), are fully or partially fixed across multiple datasets, while the scattering length density (SLD) and derived values, such as volume fraction/component coverage, can be assumed to vary. The relationships between the SLDs, layer thickness, and lipid area per molecule were defined in the software’s “custom fitting” option. Models describing the interfacial layer structure between the silicon substrate (super phase) and buffered water (subphase) consisted of a SiO_2_ layer, inner bilayer head groups, bilayer tails, and outer bilayer head groups.

NR data describing NLP_Pya_ binding to GIPC-containing SLBs were analyzed by fitting eight sets of reflectometry data describing stages of the experimental process. These were, in order, data from the following: the bare silicon surfaces in two solution isotopic contrast conditions (D_2_O and H_2_O buffers), SLB before NLP_Pya_ binding in three solution isotopic contrast conditions (D_2_O, Si-MW, and H_2_O buffers), and membrane after NLP_Pya_ binding also in three solution isotopic contrast conditions (D_2_O, Si-MW, and H_2_O buffers).

The fitting parameters for the SiO_2_ layer were assumed to be the same across all datasets obtained under varying isotopic contrast conditions of the solutions. The reflectivity datasets obtained for the silicon surfaces without the presence of the bilayer at the bulk interface were used to constrain the fitting of the roughness of the silicon and surface SiO_2_ layer. Roughness was modeled using a series of unroughened 1-Å layers (analogous to partitioning a curve for a midpoint Riemann sum) with an SLD that followed the gradient of the graded interface.

The lipid bilayer data were fitted to three parameters: the area per lipid molecule, explicitly associated water (with the lipid head group regions), and surface coverage. This strategy was useful for the membrane studies described here because it enabled the linking of the lipid head group and tail parameters, as they are part of the same molecule. This approach minimized the number of free parameters in the model and yielded useful quantities, such as hydration or area per molecule. The procedure was previously described in detail by Hughes *et al.* (*61*). Briefly, in the custom fitting script, the relationship between layer thickness, component volume, and area per molecule is defined as

Thickness=Volume/Area per molecule (1)

Component volume, scattering length (∑b), and SLD are

SLD (ρ)=∑b/Volume (2)

The volume and scattering lengths of each of the interfacial components were known or calculated. As all the membranes studied had composite lipid compositions, the molecular volume and scattering lengths used were the sum of individual lipid components multiplied by their volume fraction in the vesicles used to deposit SLBs (table S3). By fitting the interfacial lipid area per molecule, the layer thicknesses were calculated within the software and used in the matrix calculation that was used to model the reflectivity profiles and fit these to the experimental data.

Upon the interaction of NLP_Pya_, the lipid component of the membrane was again fitted using an area per molecule approach but with the addition that the nonlipid component of the membrane was described as a ratio of protein and solution. This approach allowed the volume fraction of lipid, solution, and protein in the membrane to be resolved. Lipid area per molecule, protein, and solution content of the NLP_Pya_-bound membrane was fitted independently of the initially characterized bilayer. The substrate roughness and SiO_2_ layer characteristics were constrained across all datasets. An additional layer was added to the NLP_Pya_-bound membrane, which described a layer of protein on the surface of the membrane. This layer was unconstrained, meaning that it could have a minimal thickness of 0 Å and a hydration between 0 and 100% solution (i.e., no protein). A single protein layer with asymmetric roughness on the membrane surface was found to adequately describe the protein distribution in all cases of NLP_Pya_ binding to the GIPC-containing membrane surfaces as was seen through optimal fitting of the experimental reflectometry data using this description of the protein component.

Error estimation was performed using Rascals Bayesian error estimation routines (*62*, *63*), with the log-likelihood function described in terms of chi-squared (*62*, *63*). Marginalized posteriors were obtained using a Delayed Rejection Adaptive Metropolis algorithm (*64*, *65*), and the best-fit parameters were taken as the distribution maxima; the uncertainties presented here are from the shortest 65% confidence intervals of each distribution.

The layer thicknesses and component volume fraction parameters were either intrinsically fitted (such as the SiO_2_ and protein components) or determined from the fitted area per molecule and hydration parameters (lipid component); the model fits were used to produce volume fraction versus distance profiles for the interfacial structural components. Monte Carlo Markov Chain (MCMC) Bayesian error estimation results from the model-to-data fits were used to determine ambiguity of each volume fraction versus distance profile and are shown as a line width across each component distribution. The volume fraction of an individual component was calculated in 1-Å increments from −10 to 110 Å across the solid/liquid interface (the silicon/silicon dioxide interface set as the zero position). The mean, lower, and upper 95% confidence interval bounds of each component distribution were determined for every 1-Å segment within the set range using the MCMC error estimation results; these confidence intervals were then used to produce a line width error region above and below the mean values. The water distribution was calculated as the remaining unoccupied volume and summed across the interface with the appropriate error propagation.

### Transmission electron microscopy

LUVs composed of POPC:GIPC:sterols 1:6:3 in buffer [20 mM MES and 20 mM NaCl (pH 5.8)] were incubated with 500 nM NLP_Pya_ for 5 min at 20°C. The sample suspensions (4 μl) were then applied onto Formvar-coated, carbon-stabilized, and glow-discharged (EM ACE200, Leica Microsystems, Vienna, Austria) copper grids and contrasted with 1% (w/v) uranyl acetate (aqueous solution). Samples were imaged with a CM 100 transmission electron microscope (Philips, The Netherlands) at 80 kV, using an Orius SC200 camera and Digital Micrograph Suite software (Gatan, USA).

### HS-AFM imaging

For HS-AFM imaging, SLBs were prepared by incubating 1.5 μl of 1 mM unilamellar vesicles [prepared from MLVs as described in the “Liposome preparation” section; buffer: 20 mM MES and 150 mM NaCl (pH 5.8)] on a 1.5-mm-diameter mica disk at 42°C for more than 30 min or at 55°C for less than 30 min; this was followed by extensive rinsing. HS-AFM imaging was performed with NANOEXPLORER (Research Institute of Biomolecule Metrology Co. Ltd., Tsukuba, Japan) using cantilevers with carbon nanofiber probes (BL-AC10FS-A2) provided by Olympus Co. (Tokyo, Japan). The cantilevers had a spring constant of 0.1 N/m and a resonance frequency of 450 to 500 kHz in aqueous medium. The scan direction for image acquisition was from left to right, and the scan rate was 0.5 frame/s. The SLB-deposited mica surface was imaged in 70 to 80 μl of buffer solution. Experiments were performed in 20 mM MES (pH 5.8) with or without 150 mM NaCl. After observing SLB, 10 μl of NLP_Pya_ was introduced into the imaging medium. The final concentration of NLP_Pya_ in the imaging medium was 300 nM or 10 μM.

### Planar lipid bilayer experiments

Experiments were performed with the Nanion Technologies Orbit Mini set up and Ionera 100-μm MECA recording chips. Data were obtained with the Elements Data Reader v 3.8.3 (Elements) software package at a working range of 2 nA, sampling frequency of 20 kHz, and room temperature. DPhPC, GIPCs, and sterols were dissolved to 5 mg/ml in pure octane (analytical standard, Sigma-Aldrich Inc., St. Louis, MO, USA). Bilayers were formed by the manufacturer-provided brush. Recordings were performed in 120 μl of recording buffer [20 mM MES (pH 5.8), with 0.1 or 1 M NaCl] at −100 mV (for NLP_Pya_) or 50/100 mV (for the lysenin mutant RL1 pore) (*21*). NLP_Pya_ and lysenin were tested at 9.4 to 20.6 μM and 0.5 to 48.8 pM, respectively. Data were analyzed using Axon pCLAMP 11.1 software and filtered using a Butterworth (eight-pole) low-pass filter with a cutoff frequency of 1000 Hz.

### GUV preparation

GUVs used in the microfluidic and dextran leakage experiments were prepared with a modified electroformation method (*22*). For the microfluidic experiments, 20 ml of POPC:GIPC 1:1.75 (n:n) mixture (1 mg/ml) was spread over two platinum electrodes and dried in vacuum for at least 4 hours. The electrodes were inserted into 2-ml Eppendorf tubes, which were filled with 2 ml of 0.2 M sucrose and 5 μM A594 (molecular weight, 758.79 g/mol; Molecular Probes, Invitrogen, Thermo Fisher Scientific) solutions. The tubes were placed in a water bath at 65°C, and the electrodes were connected to an AC generator set to an amplitude of 8 V and a frequency of 10 Hz for the first 2 hours. After 2 hours, the water heating was switched off, and the settings were gradually lowered to 1 V and 1 Hz. The suspension of formed vesicles was cooled down to room temperature for ~30 min and then transferred into a plastic container together with an equal amount of an iso-osmolar glucose solution. The denser, sucrose-filled GUVs sank in the less dense sucrose/glucose mixture. The container with the GUV suspension was sealed, and the vesicles were used within 4 days.

GUVs [POPC:GIPC 1:1.2 (n:n)] for the dextran leakage experiments monitored by confocal laser scanning microscopy were prepared on a commercially available vesicle preparation station (Vesicle Prep Pro station, Nanion Technologies, Germany). Lipids were dissolved in chloroform:methanol 9:1 (v:v) to 2.5 mM, and the fluorescent probe rhodamine-DHPE was added for membrane labeling at a final lipid concentration of 0.5%. Lipid solution (10 μl) was placed on the conductive indium tin oxide–coated glass slide and dried under reduced pressure for at least 60 min. The lipid-covered slide was then transferred onto the vesicle preparation station, with dried lipid film positioned in the center of the O-ring. The lipid film was rehydrated in 750 μl of ultrapure water at 55°C and covered with another conductive slide. Electroformation was performed inside the station with an AC current of 3.5 V and 10 Hz applied across the slides for 165 min at 65°C. This procedure continued for another 75 min at 22°C and 0.5-V amplitude. GUVs sedimented spontaneously to the bottom of the conical falcon tube in ~4 days.

### Confocal laser scanning microscopy

For the NLP_Pya_ permeabilization experiments, freshly prepared GUV suspensions were mixed with 4-, 10-, or 70-kDa FDs (Sigma-Aldrich, St. Louis, MO, USA) and NLP_Pya_. The final concentrations of FDs and NLP_Pya_ were 1 mg/ml and 500 nM, respectively. The mixture in ultrapure water was incubated for 30 min at room temperature. Images were then recorded on a Leica TCS SP5 laser-scanning microscope with a 63× oil-immersion objective (numerical aperture = 1.4), with fluorescence excitation/emission λ ranges of 480/500 to 530 nm (for FDs) and 543/644 to 650 nm (for rhodamine). Acquired images were analyzed with ImageJ software (ImageJ 1.29×, NIH, USA), and the permeabilization (%) of individual vesicles was calculated.

### Microfluidic experiments

The real-time interaction of NLP_Pya_ labeled with A488 (Molecular Probes, Invitrogen, Thermo Fisher Scientific) (NLP_Pya_-A488) with individual GUVs was studied with a microfluidic system with a diffusion chamber (fig. S14A). The design, fabrication, and characteristics of the microfluidic system were described previously (*22*). Briefly, the main microchannel in the microfluidic chip is 100 μm wide and 40 μm deep. The flow in the main channel can be hydraulically regulated by a simple adjustment of the height of the exit reservoir, and the solution that flows through the system can be easily exchanged in the entrance reservoir. The diffusion chamber (100 μm × 250 μm × 40 μm) is a dead-end channel extending sideways from the main channel. The chamber is essentially flow-free, and therefore, the exchange of solutes between the main channel and diffusion chamber only occurs via passive diffusion (*66*).

The microfluidic system was set on an inverted microscope (Nikon Eclipse Ti) equipped with an epifluorescence module (pE-300ultra, CoolLED, UK), optical tweezers (Tweez, Aresis, Slovenia; laser wavelength of 1064 nm), and a 60× water immersion objective (NIR APO; numerical aperture, 1.0). Experiments were recorded with a sCMOS camera (Zyla 5.5, Andor, UK) and analyzed with ImageJ (*67*) and a custom-made MatLab software.

A fresh microfluidic system was prepared for each set of experiments. The system was first flushed with an iso-osmolar glucose solution. A suspension of GUVs was introduced into the main channel, and the flow rate was set to approximately 10 μm/s. Suitable GUVs without visible membrane protrusions were selected from the main channel and individually transferred by optical tweezers into the diffusion chamber. Then, the system was flushed with an iso-osmolar solution without a fluorescent marker so that any fluorescent signals present originated only from A594 trapped inside GUVs. Last, an iso-osmolar solution of sucrose or glucose with 1 to 1.3 μM NLP_Pya_-A488 was introduced into the main channel and diffused toward GUVs in the diffusion chamber. Since the diffusion was slow, the protein concentration gradient along the chamber persisted throughout the experiment (the concentration at the rear end of the chamber was always lower than that in the main channel). The experiments were recorded in 3-s time lapses in transmitted light (to monitor membrane morphology) as well as in green (for NLP_Pya_-A488) and red (for A594) epifluorescence channels. As the protein diffused toward GUVs, some GUV movement was observed (movie S4) that could be diffusiophoretic in origin (*68*).

For each GUV, the intensities of three fluorescent signals were recorded: A594 in the vesicle interior, NLP_Pya_-A488 in the solution surrounding GUV, and NLP_Pya_-A488 on the GUV membrane. All fluorescent signals were corrected for interchannel cross-talk and fluorescent marker bleaching. The NLP_Pya_-A488 signal on the membrane was determined as the difference between the average intensity on the membrane minus the intensity in the surrounding solution. The A594 signal in the vesicle was calculated as the average A594 intensity in the vesicle interior.

To quantify the dynamics of NLP_Pya_ binding and membrane permeabilization for each vesicle, the signals of NLP_Pya_-A488 on the membrane and A594 inside the vesicles were fitted by a logistic sigmoidal curve (fig. S14B). The time intervals of the NLP_Pya_-A488 signal increasing on the membrane (τ_bind_), the time lag between the saturation of the signal on the membrane and the onset of A594 leaking (τ_lag_), and the time interval during which A594 molecules diffused out of the vesicle after the membrane has been permeabilized (τ_leak_) were quantified by fitting sigmoidal curves to the fluorescence signals (dashed black curves on fig. S14B) and calculating the times when the fitted curves reached thresholds at 10 and 90%. τ_bind_ for the binding of the protein to the membrane was determined as the difference between the times where the sigmoid for NLP_Pya_-A488 reached 10 and 90% of its final value. Here, it should be noted that the increasing of the NLP_Pya_-A488 signal on the membrane is coupled to the diffusion of NLP_Pya_ into the diffusion chamber, while the partition of NLP_Pya_ from the surrounding solution to the vesicle membrane is rapid. τ_lag_ was determined as the difference between the time when the sigmoid for NLP_Pya_-A488 reached 90% of its final value and the time when the sigmoid for A594 reached 90% of its initial value. τ_leak_ was determined as the difference between the times when the sigmoid for A594 decreased from 90 to 10% of its initial value. A total of 21 GUVs were analyzed in six independent experiments. In one of the analyzed GUVs, positioned at the very rear of the chamber where the NLP_Pya_-A488 concentration was the lowest, A594 leakage was not observed during the entire experiment (~1 hour) despite visible NLP_Pya_-A488 binding. In control experiments, in which GUVs were exposed to an iso-osmolar solution without the protein, GUVs remained stable in shape and without leakage of A594 for more than 2 hours.
