# Supplementary material for: An oomycete NLP cytolysin forms transient small pores in lipid membranes
Source: Sci Adv. 2022 Mar 11;8(10):eabj9406. doi: 10.1126/sciadv.abj9406 (PMC8916740; doi:10.1126/sciadv.abj9406)
Supplement: Supplementary file 1 — Supplementary Text S1 Figs. S1 to S16 Tables S1 to S3 [file sciadv.abj9406_sm.pdf]

Science Advances
AAAS

Supplementary Materials for

An oomycete NLP cytolysin forms transient small pores in lipid membranes

Katja Pirc, Luke A. Clifton, Neval Yilmaz, Andrea Saltalamacchia, Mojca Mally, Tina Snoj, Nada Žnidaršič, Marija Srnko, Jure Borišek, Petteri Parkkila, Isabell Albert, Marjetka Podobnik, Keiji Numata, Thorsten Nürnberger, Tapani Viitala, Jure Derganc, Alessandra Magistrato, Jeremy H. Lakey, Gregor Anderluh*

*Corresponding author. Email: gregor.anderluh@ki.si

Published 11 March 2022, *Sci. Adv.* **8**, eabj9406 (2022)
DOI: 10.1126/sciadv.abj9406

The PDF file includes:

Supplementary Text S1
Figs. S1 to S16
Tables S1 to S3
Legends for movies S1 to S4

Other Supplementary Material for this manuscript includes the following:

Movies S1 to S4

Supplementary Text S1.
Modeling of the fluorescent signals in the microfluidic experiments

Microfluidic experiments yield data for the membrane-bound fluorescently labeled NLP protein (NLP_{Pya}-A488), $c_{NLP}(t)$, and the concentration of the fluorescent dye A594 in the giant unilamellar vesicle (GUV) interior, $c_{A594}(t)$. Here we use these data to test two possible models of NLP_{Pya}-induced membrane leakage from a GUV.

The A594 leakage depends on the following variables and parameters:

$c_{NLP}(t)$	concentration of membrane-bound NLP_{Pya} (this is a measured parameter, corresponding to the intensity of A488, here expressed in dimensionless arbitrary units)
$c_{A594}(t)$	concentration of A594 in the vesicle (this is a measured parameter, normalized to the maximal intensity of the A594 signal, here expressed in dimensionless arbitrary units)
$c_{mono}(t)$	concentration of NLP_{Pya} monomers on the membrane (dimensionless units)
$c_{cl}(t)$	concentration of clustered NLP_{Pya} on the membrane (dimensionless units)
$P(t)$	membrane permeability (in (m/s))
R	GUV radius (in (m))
A	GUV surface area ($A = 4\pi R^2$)
V	GUV volume ($V = 4\pi R^3 / 3$)

a) Leakage of A594 from the vesicle

Assuming that c_{A594} inside the vesicle is homogeneous (i.e., that diffusion of A594 within the vesicle is much faster than the leakage of A594 out of the vesicle), $c_{A594}(t)$ can be described by a standard equation for solute leakage:

$$\frac{dc_{A594}(t)}{dt} = -\frac{P(t)A}{V} c_{A594}(t) = -\frac{3P(t)}{R} c_{A594}(t) \qquad (1)$$

b) Relation between membrane permeability and membrane-bound NLP_{Pya}

Simple model assuming that membrane permeability is proportional to the membrane-bound NLP_{Pya}

Within this model, the membrane permeability can be written as

$$P(t) = k_p\, c_{NLP}(t), \qquad (2)$$

where k_p is a membrane permeability coefficient (in (m/s)). In this case, the concentration of A594 inside the vesicle follows a simple kinetic equation:

$$\frac{dc_{A594}(t)}{dt} = -\,K_p\,c_{NLP}(t)\,c_{A594}(t), \tag{3}$$

where K_p is the only kinetic constant for A594 leakage (it is related to the size of the vesicle and the permeability coefficient, $K_p = 3\,k_p / R,$ in (s^{-1})). Within this model, the A594 starts leaking as soon as the protein concentration on the membrane increases (the value of K_p only affects the slope of the leakage curve, i.e. the leaking rate) and thus the model cannot account for the observed time-lag (fig. S15A). In addition, the time-lag cannot be reproduced even if one assumes that membrane permeability depends on cooperative action of NLP_{Pya} proteins on the membrane, i.e., if

$$P(t) = k_p\,c_{NLP}^{n}(t), \tag{4}$$

where n is the number of participating NLP_{Pya} monomers in an induced pore.

A minimal two-step model of slow nucleation and fast auto-catalytic growth

We continue with the assumption that NLP_{Pya} clusters have to form in order for the membrane to become permeable. Assuming that membrane permeability $P(t)$ is proportional to the amount of NLP_{Pya} clusters in the membrane

$$P(t) = k_p\,c_{cl}(t), \tag{5}$$

the concentration of A594 can be calculated from:

$$\frac{dc_{A594}(t)}{dt} = -\,K_p c_{cl}(t)\,c_{A594}(t). \tag{6}$$

We then assume that NLP_{Pya} clusters form from NLP_{Pya} monomers through a 2-step Finke-Watzky minimalistic model of nucleation and growth (*26*). According to this model, the concentration of clusters follows:

$$\frac{dc_{cl}(t)}{dt} = k_1 c_{mono}(t) + k_2 c_{mono}(t)\,c_{cl}(t), \tag{7}$$

where k_1 and k_2 correspond to nucleation and assembly growth rates in (s^{-1}), respectively (*25*). In this case, the sum of the amounts of clusters and monomers equals the total amount of the membrane-bound NLP_{Pya}, which is proportional to the measured fluorescence signal:

$$c_{mono}(t) + c_{cl}(t) = c_{NLP}(t). \tag{8}$$

As presented in fig. S15B, this model readily describes both the time-lag and the rate of the A594 leakage.

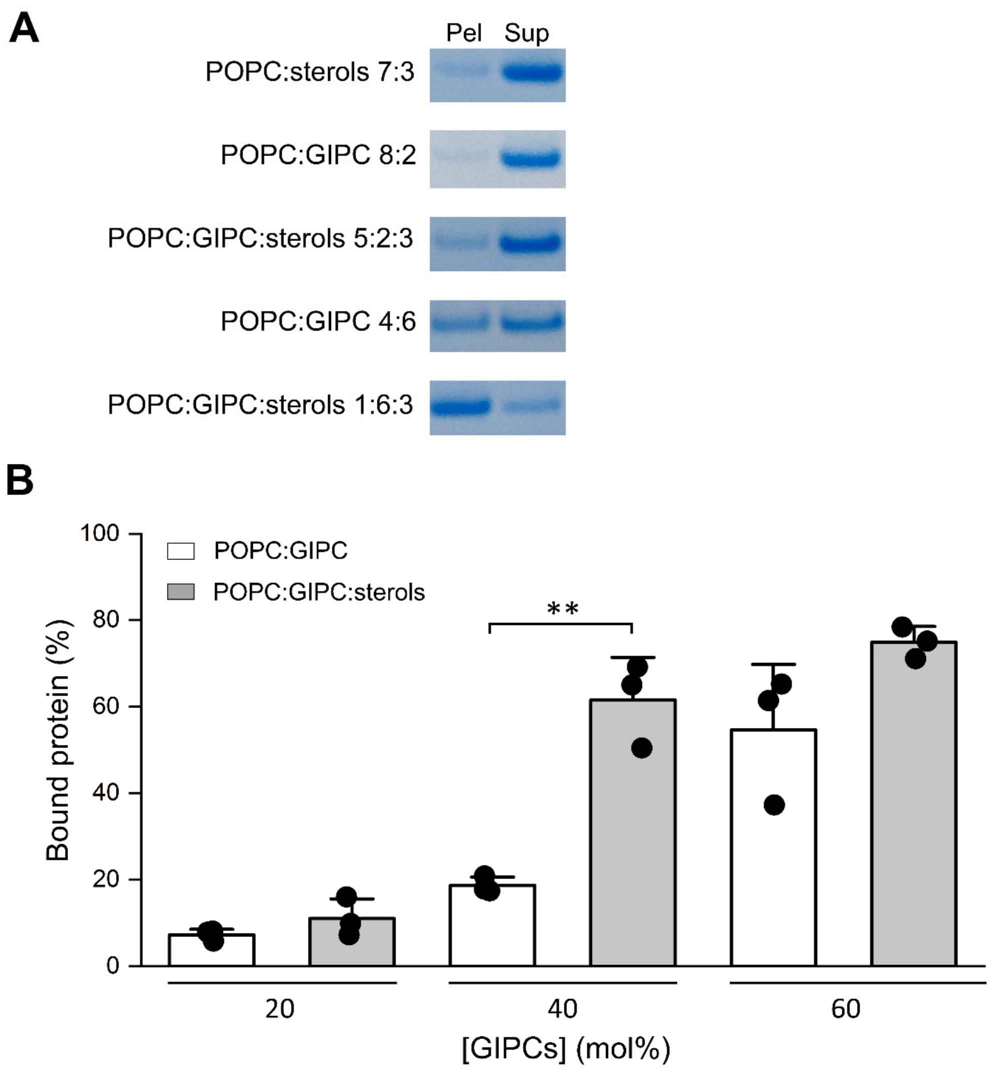

Fig. S1.
The effect of membrane composition on the binding of NLP_{Pya} as revealed by liposome sedimentation. (**A**) The binding of NLP_{Pya} to multilamellar vesicles with specific lipid compositions. Pel, pellet; Sup, supernatant. (**B**) The quantification of NLP_{Pya} binding from Fig. 1A and (A). The concentration of GIPCs in the vesicles varied (as indicated), while sterols represented 30 mol%. Buffer: 20 mM MES and 150 mM NaCl, pH 5.8. Values are means ± SD (n=3), analyzed with the Student's *t*-test (**$P < 0.01$).

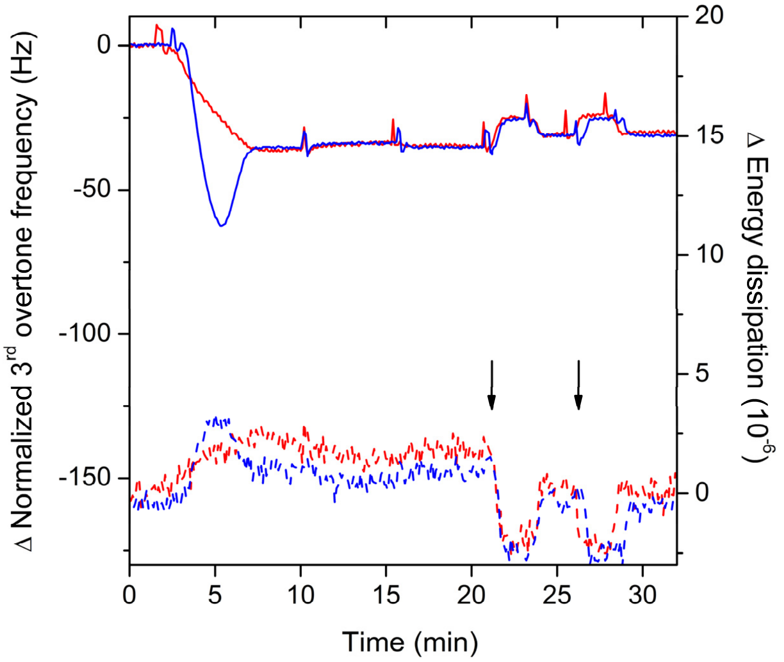

Fig. S2.

Supported lipid bilayer (SLB) formation during the quartz crystal microbalance experiments. SLBs composed of POPC:GIPC 6:4 (red) and POPC:GIPC:sterols 3:4:3 (blue) formed from small unilamellar vesicles. The solid lines show the normalized changes in the 3rd overtone frequency. Dashed lines correspond to the changes in energy dissipation, which can be used to assess the quality of the SLBs. The return of the energy dissipation to baseline after the osmotic shock induced by the ultrapure water additions (indicated by arrows) suggests that all the vesicles ruptured and formed SLBs with minor defects. Buffer: 20 mM MES and 150 mM NaCl, pH 5.8.

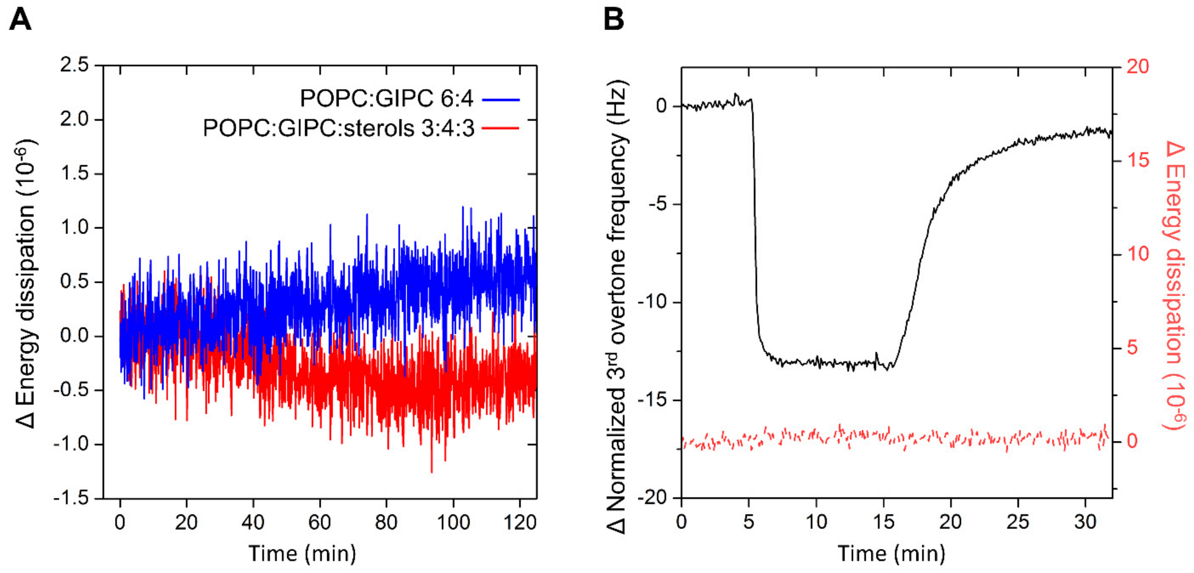

Fig. S3.

The binding of NLP_{Pya} to the supported lipid bilayers during the quartz crystal microbalance experiments. (**A**) A change in energy dissipation (3rd overtone) during the kinetic titration experiments presented in Fig. 1B. (**B**) Binding of 40 µM NLP_{Pya} to the supported lipid bilayer composed of POPC:GIPC:sterols 3:4:3. GIPCs were purified from tomato leaves. The change in normalized 3rd overtone frequency is proportional to the bound surface-mass density. The negligible change in energy dissipation indicates that NLP_{Pya} (40 µM) does not influence the viscoelastic properties of the bilayer. Buffer: 20 mM MES and 150 mM NaCl, pH 5.8.

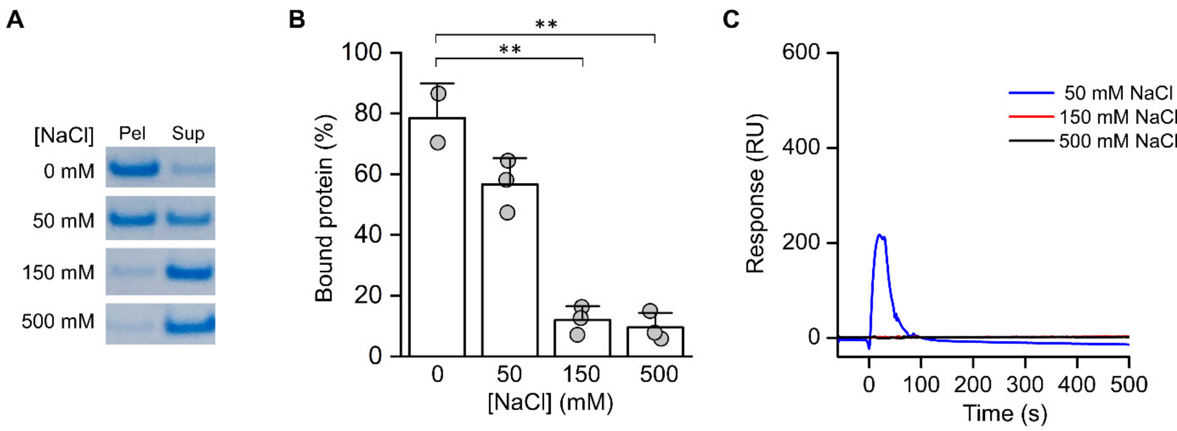

Fig. S4.
The effect of NaCl concentration on the binding of NLP_{Pya} to negatively charged phospholipids. (**A**) The binding of NLP_{Pya} to POPC:POPG 6:4 multilamellar vesicles at different NaCl concentrations, monitored by sedimentation assay. Pel, pellet; Sup, supernatant. (**B**) The quantification of the binding of NLP_{Pya} to vesicles from (A). Values are means ± SD (n=3), analyzed with the Student's *t*-test (**$P < 0.01$). (**C**) Surface plasmon resonance analysis of NLP_{Pya} binding to immobilized large unilamellar vesicles composed of POPC:POPG 6:4 at different NaCl concentrations. Buffer: 20 mM MES, 2 mM EDTA, and different NaCl concentrations, pH 5.8.

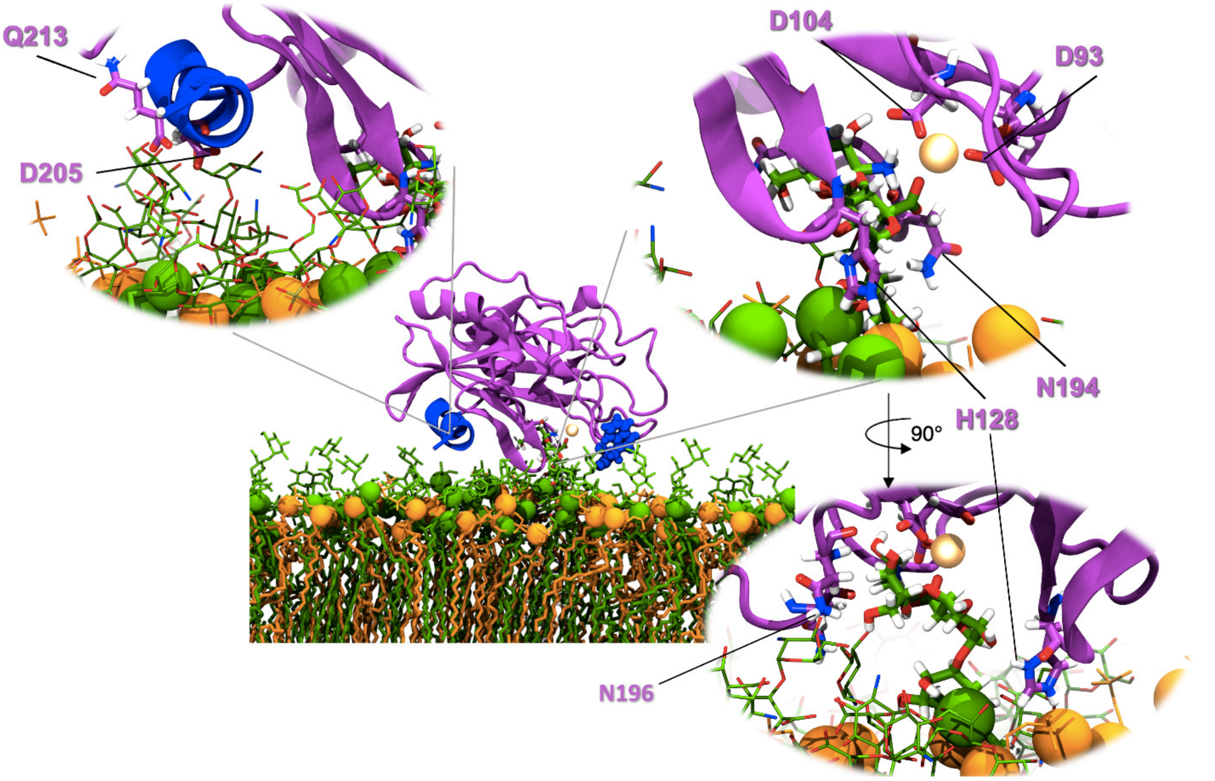

Fig. S5.
The hydrogen bonding network at the membrane interface. The scheme displays the following: POPC lipids (orange carbon atoms), GIPC lipids (green carbon atoms), phosphate groups (van der Waals spheres), NLP_{Pya} (magenta), the residues establishing hydrogen-bonding interactions with the membrane (magenta carbon atoms), oxygen (red), hydrogen (white), nitrogen, the tryptophan 155 (W155; presented as van der Waals spheres) and the C-terminal helix (blue), Mg^{2+} (gold van der Waals spheres). The three insets show a magnified view of the NLP_{Pya} interactions with the membrane and at the C-terminal helix (left panel) and at the binding cavity (right top and bottom panel). NLP_{Pya} binding is mostly stabilized by electrostatic interactions (i.e. the electrostatic and van der Waals components of total interaction energy between NLP_{Pya} and the membrane are of -240.9 ± 5.7 and -57.4 ± 4.5 kcal/mol, respectively).

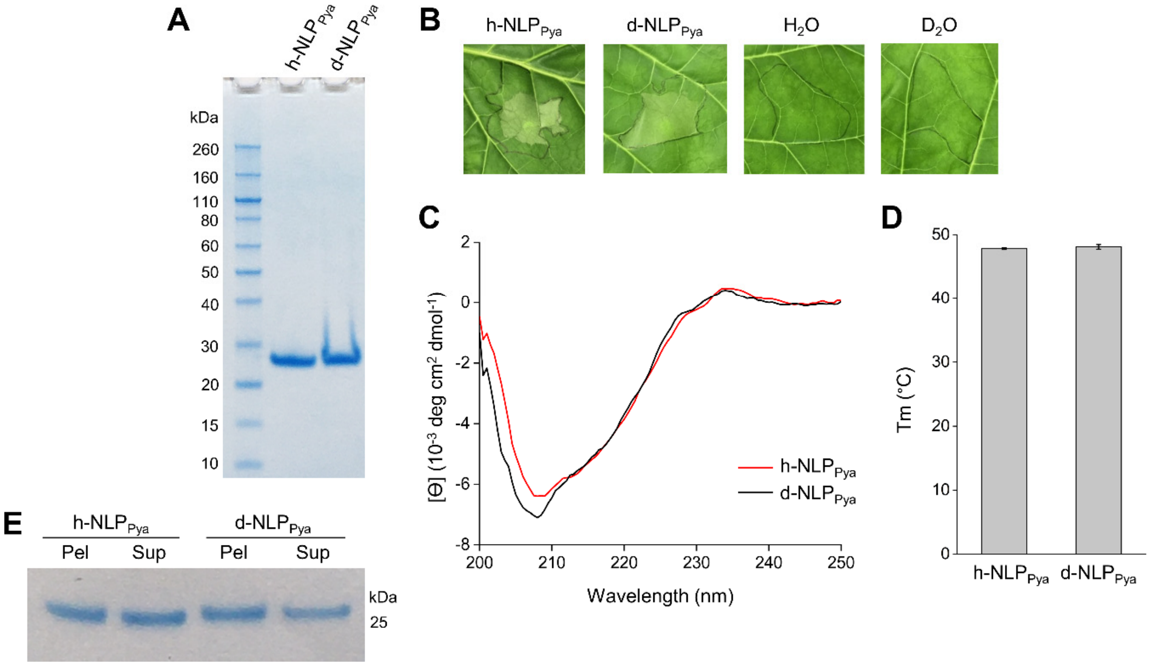

Fig. S6.

Comparative analysis of h-NLP$_{Pya}$ and d-NLP$_{Pya}$. (**A**) SDS-PAGE of the recombinant proteins. (**B**) Leaf infiltration assays indicate comparable necrotic activities of the proteins. Circular dichroism (**C**) and differential scanning fluorimetry (**D**) reveal comparable secondary structural properties and stabilities of both proteins. (**E**) Liposome sedimentation assay confirmed comparable binding to multilamellar vesicles composed of POPC:GIPC:sterols 3:4:3. Pel, pellet; Sup, supernatant. Buffer: 20 mM MES, 50 mM NaCl, and 2 mM EDTA, pH 5.8, in H_2O. Experiments were repeated three times with similar results.

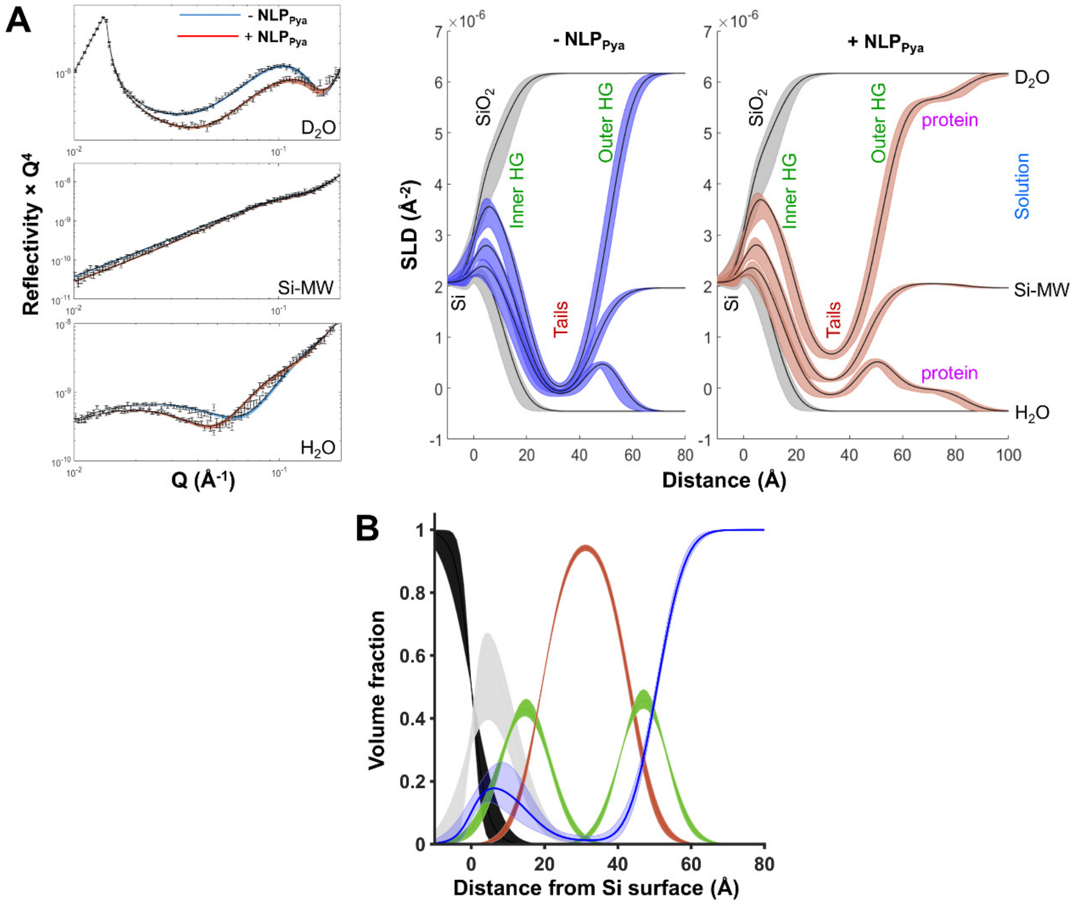

Fig. S7.

Neutron reflectivity data, scattering length density (SLD) profiles, and component volume fraction *vs.* distance profiles for POPC:GIPC 6:4 supported lipid bilayers in the absence or presence of h-NLP$_{Pya}$. (**A**) Reflectometry data for each isotopic contrast (left) and the SLD profiles (right) before and after the interaction of the protein. The shaded areas in both the model-to-data fits and SLD profiles depict the 65% confidence intervals of the model parameters determined from Monte Carlo resampling of the experimental data fits. Gray profiles were obtained from the neutron reflectivity data from the silicon surface prior to the deposition of the supported lipid bilayers. (**B**) Component volume fraction *vs.* distance profiles determined from the analysis of neutron reflectivity data of supported lipid bilayers before the interaction of the protein. The following is displayed: substrate layer distributions (silicon (black) and silicon dioxide (gray)), lipid head groups (green), lipid tails (red), and water distribution across the membrane (blue). Line widths depict the ambiguity in component position and volume fraction as 65% confidence intervals of the acceptable parameter ranges determined from Monte Carlo resampling of the experimental data fits in (A).

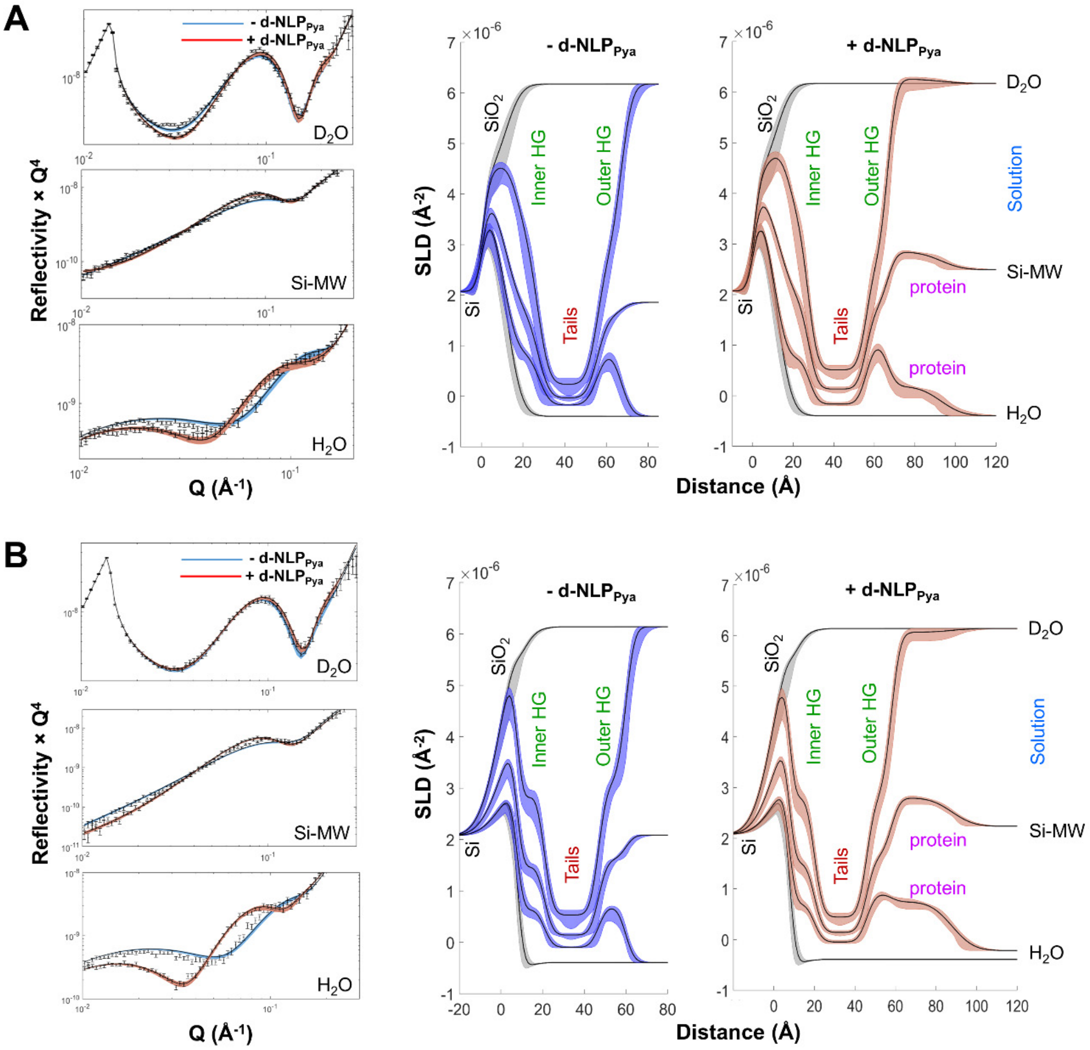

Fig. S8.
Neutron reflectivity data and scattering length density (SLD) profiles for supported lipid bilayers composed of POPC:GIPC 6:4 (A) and POPC:GIPC:sterols 3:4:3 (B) in the absence or presence of d-NLP$_{Pya}$. Reflectometry data for each isotopic contrast (left) and the SLD profiles (right) for the supported lipid bilayers before and after the interaction of the protein. The shaded areas in both the model-to-data fits and SLD profiles depict the 65% confidence intervals of the model parameters determined from Monte Carlo resampling of the experimental data fits. Gray profiles were obtained from the neutron reflectivity data from the silicon surface prior to the deposition of the supported lipid bilayers.

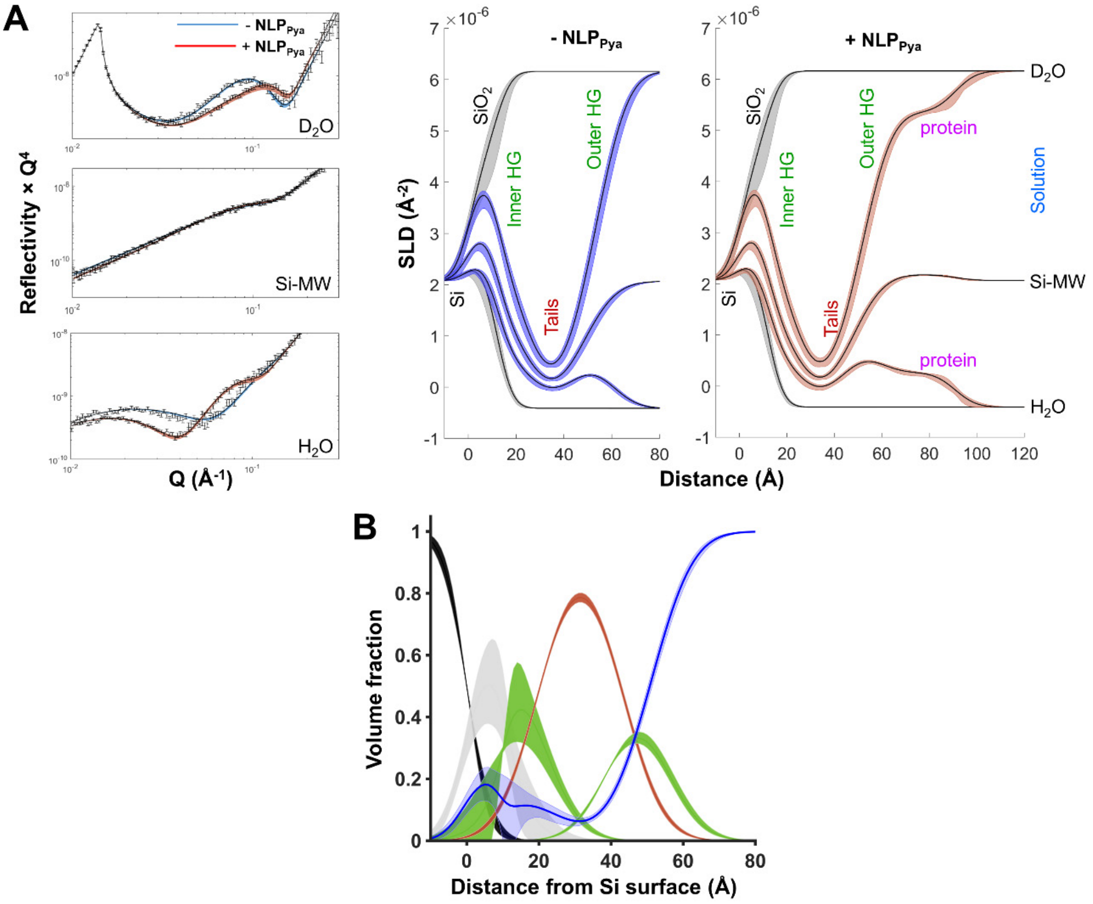

Fig. S9.

Neutron reflectivity data, scattering length density (SLD) profiles, and component volume fraction *vs.* distance profiles for POPC:GIPC:sterols 3:4:3 supported lipid bilayers in the absence or presence of h-NLP$_{Pya}$. (**A**) Reflectometry data for each isotopic contrast (left) and the SLD profiles (right) for the supported lipid bilayers before and after the interaction of the protein. The shaded areas in both the model-to-data fits and SLD profiles depict the 65% confidence intervals of the model parameters determined from Monte Carlo resampling of the experimental data fits. Gray profiles were obtained from the neutron reflectivity data from the silicon surface prior to the deposition of the supported lipid bilayers. (**B**) Component volume fraction *vs.* distance profiles determined from the analysis of neutron reflectometry data of supported lipid bilayers before the interaction of the protein. The following is displayed: the substrate layer distributions (silicon (black) and silicon dioxide (gray)), lipid head groups (green), lipid tails (red), and water distribution across the membrane (blue). Line widths depict the ambiguity in component position and volume fraction as 65% confidence intervals of the acceptable parameter ranges determined from Monte Carlo resampling of the experimental data fits in (A).

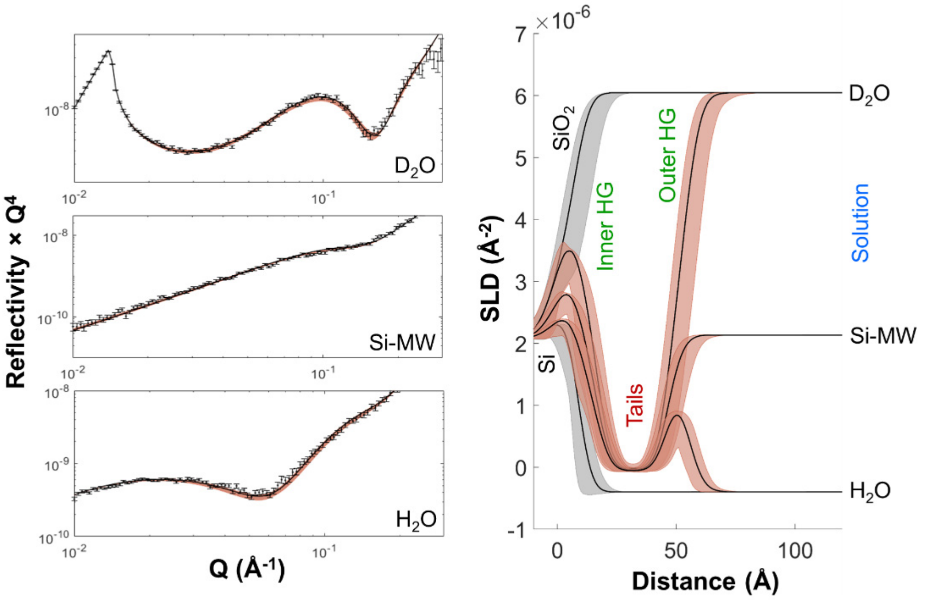

Fig. S10.
Neutron reflectivity data and scattering length density (SLD) profiles for a POPC:sterols 7:3 supported lipid bilayer after h-NLP$_{Pya}$ binding. Reflectometry data for each isotopic contrast (left) and the SLD profiles (right). The shaded areas depict the 65% confidence intervals of the model parameters determined from Monte Carlo resampling of the experimental data fits. Gray profiles were obtained from the neutron reflectivity data from the silicon surface prior to the deposition of the supported lipid bilayer.

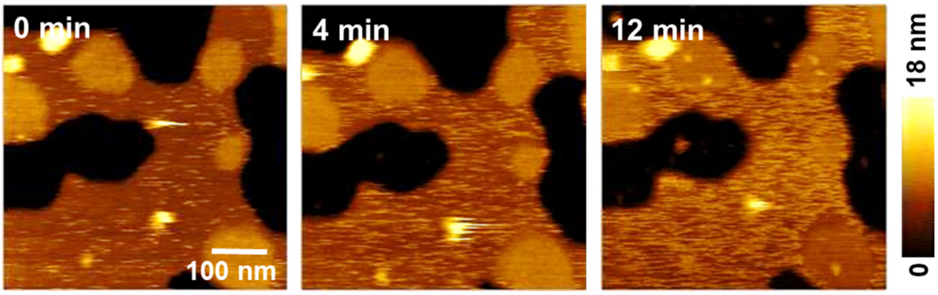

Fig. S11.
High-speed atomic force microscopy images showing the binding of NLP_{Pya} to the supported lipid bilayer composed of POPC:GIPC:sterols 1:6:3. The timepoint at which NLP_{Pya} (with a final concentration of ~300 nM) was added to the imaging medium (20 mM MES, pH 5.8) is depicted as 0 min. Black areas in the image represent mica support, and the dark and bright areas are L_d and L_o lipid phases, respectively (see also Fig. 3B).

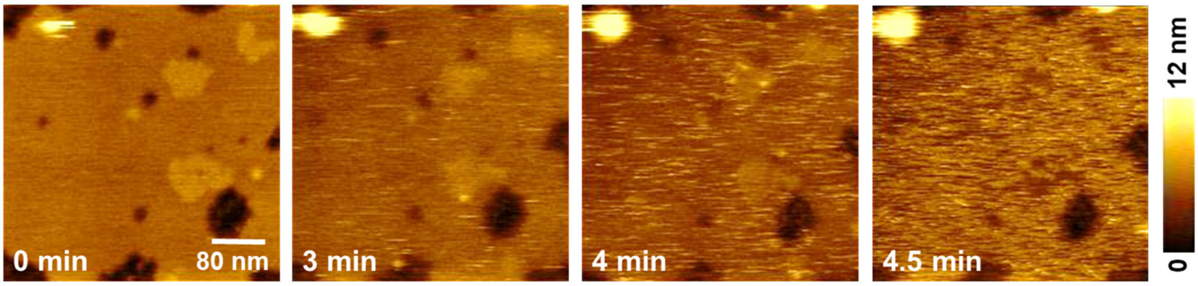

Fig. S12.
High-speed atomic force microscopy images showing the binding of NLP_{Pya} to the supported lipid bilayer composed of DOPC:GIPC:sterols 1:1:1. The timepoint at which NLP_{Pya} (with a final concentration of ~10 µM) was added into the imaging medium (20 mM MES and 150 mM NaCl, pH 5.8) is depicted as 0 min. Black areas in the image represent mica support, and the dark and bright areas are L_d and L_o lipid phases, respectively (see also Fig. 3B).

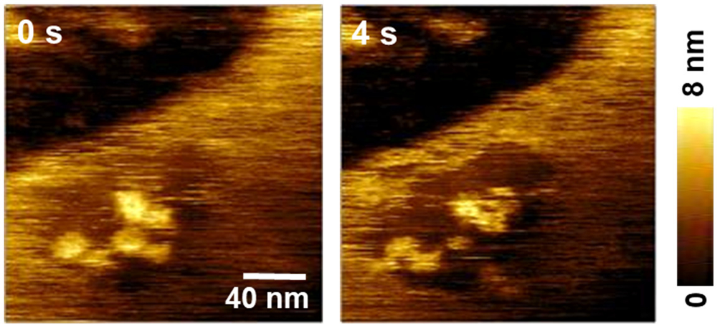

Fig. S13.
High-speed atomic force microscopy images showing NLP_{Pya} aggregates on the supported lipid bilayer composed of DOPC:GIPC:sterols 1:6:3. NLP_{Pya} aggregates at 0 s and 4 s. Imaging buffer: 20 mM MES, pH 5.8. The concentration of NLP_{Pya} was 300 nM.

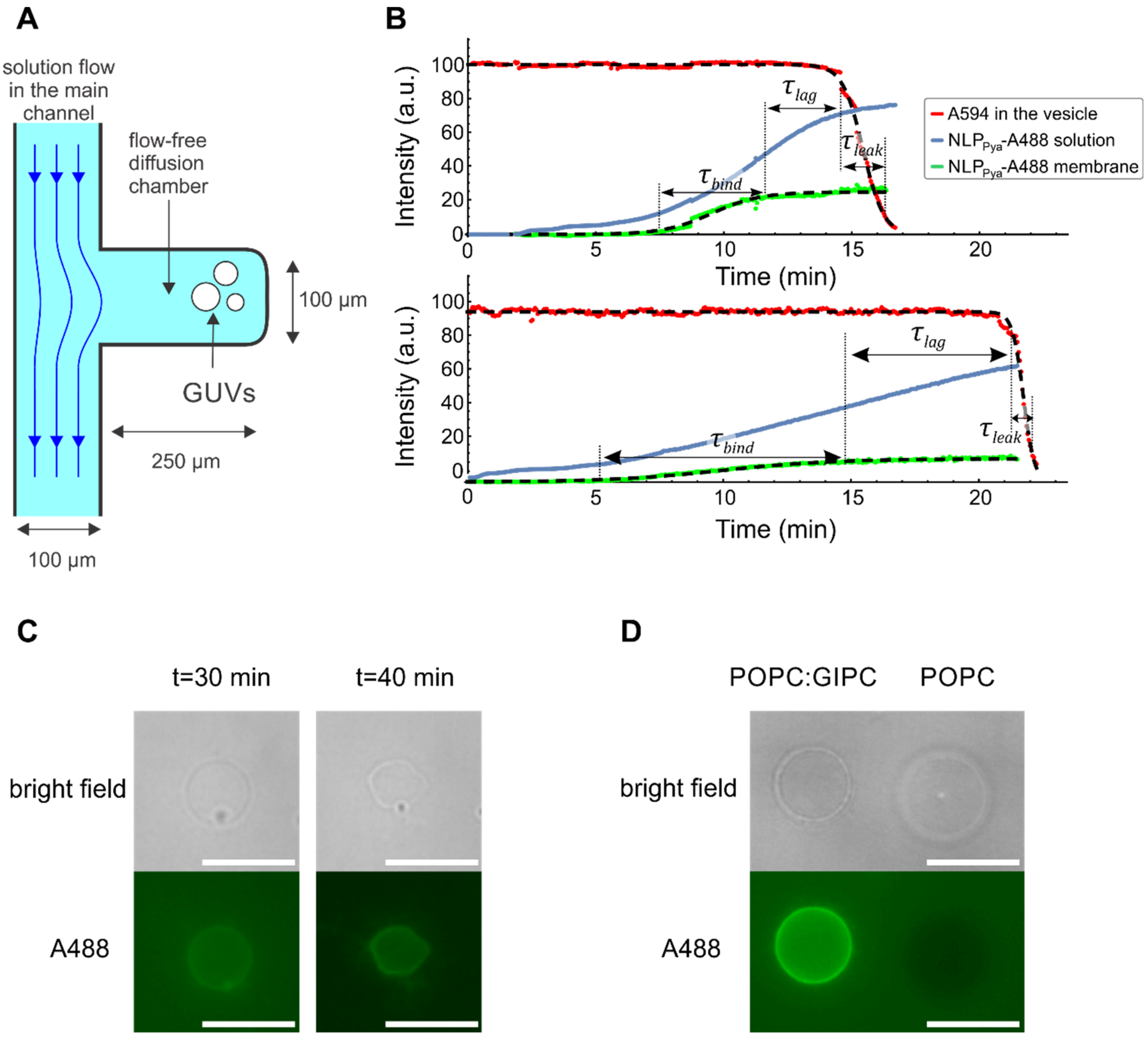

Fig. S14.
The microfluidic chamber for the simultaneous monitoring of the binding of NLP_{Pya} and membrane permeabilization. (**A**) A schematic representation of the microfluidic system with a main channel and a diffusion chamber. The vesicles are transferred into the chamber from the main channel with optical tweezers. The solution in the main channel can be exchanged by emptying and refilling the entrance reservoir, whereas the solutes enter the chamber solely via diffusion. (**B**) The time-dependence of fluorescent signals for two representative vesicles filled with A594 and exposed to NLP_{Pya}-A488 at 1.3 µM (top) and 1 µM (bottom). The time intervals of the NLP_{Pya}-A488 signal increasing on the membrane (τ_{bind}), the time-lags between the saturation of the NLP_{Pya}-A488 signal on the membrane and the onset of leaking (τ_{lag}), and the time intervals of A594 leakage (τ_{leak}) are depicted. (**C**) Bright field and fluorescence (A488) images of a POPC:GIPC GUV before (t = 30 min) and after (t = 40 min) NLP_{Pya}-A488 was washed away from the diffusion chamber (see movie S4). Scale bars: 20 µm. (**D**) Bright field and fluorescence (A488) images of POPC:GIPC and POPC GUVs placed side-by-side in the diffusion chamber in a single experiment and exposed to NLP_{Pya}-A488 for ~1 h. Scale bars: 20 µm.

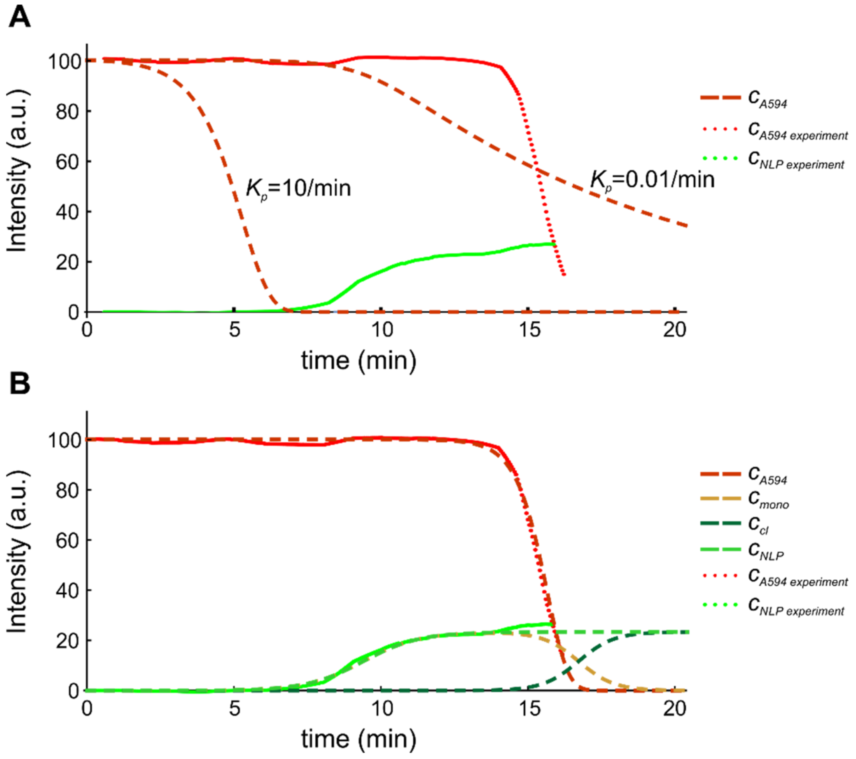

Fig. S15.

A representative example of model fitting to the data of A594 leakage for a vesicle presented in Fig. 5B. (**A**) Kinetics of A594 leakage assuming that the membrane permeability is proportional to the concentration of membrane-bound NLPPya for two values of the permeability constant (K_p = 0.01 min^{-1} and K_p = 10 min^{-1}). Neither of these limits can account for the observed time-lag followed by fast leakage. Red and green dots are the measured signals of A594 in the vesicle and NLPPya-A488 on the membrane, respectively, and the model results are represented by dashed curves. (**B**) Kinetics of A594 leakage assuming a minimal two-step model, where a slow nucleation process of NLPPya monomers on the membrane is followed by fast auto-catalytic assembly of pore-forming clusters. The permeability constant K_p was set to 1 min^{-1}, and accordingly the kinetic constants were k_1 = 0.000009 min^{-1} and k_2 = 0.17 min^{-1}. Red and green dots are the measured signals of A594 in the vesicle and NLPPya-A488 on the membrane, respectively, and the model results are represented by dashed curves. Legend: c_{A594}, concentration of A594 in the vesicle; c_{NLP}, concentration of membrane-bound NLPPya; c_{mono}, concentration of NLPPya monomers on the membrane; c_{cl}, concentration of clustered NLPPya on the membrane.

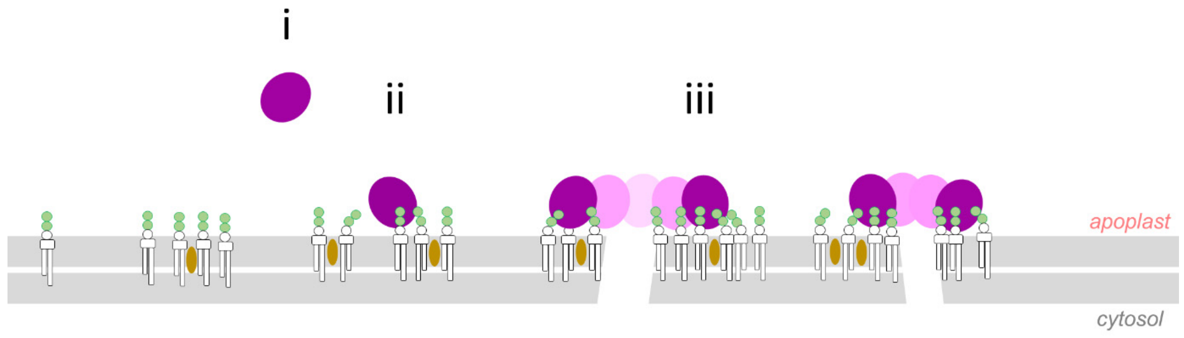

Fig. S16.

A model of NLP-induced plant membrane damage. Soluble NLP_{Pya} (i) binds to plant plasma membrane by specifically recognizing negatively charged GIPCs in the membrane (ii). The receptor binding cavity of NLP_{Pya} embraces GIPC's head group containing two terminal sugars. The protein also establishes stable interactions with several other GIPC's head groups. The initial interaction with the plant membrane is predominantly electrostatically driven. NLP_{Pya} further assembles into aggregates that are heterogeneous in size and shape (iii). GIPC's very long fatty acids can have a structural role in coupling both membrane leaflets (*14*). Transient membrane ruptures occur after a membrane is saturated with NLP_{Pya}, which cause the redistribution of GIPCs due to numerous contacts with NLP_{Pya}. In this model, NLP_{Pya} resides on the membrane surface and does not penetrate deeply into the bilayer throughout the pore formation process. According to MD simulations, the regions that may be responsible for membrane rupture formation are the loops around the GIPC binding site and the C-terminal helix. Legend: NLP_{Pya} (magenta), sterols (gold), GIPC sugar head group (green), and GIPC inositol phosphorylceramide part (white).

Table S1.
Interaction energies (kJ/mol) between the NLP_{Pya} residues and membrane lipids. Individual residue contribution (electrostatic (El) and hydrophobic (vdW), along with their sum (Tot)) is listed if larger than 5 kJ/mol.

NLP_{Pya} residue	El	vdW	Tot
Asp205	-120.8	-0.197	-120.997
Gln213	-116.9	7.47	-109.43
Asn194	-65.3	-12.7	-78
Lys209	-49.9	-14.6	-64.5
Asn196	-28	-15	-43
Lys152	-28.2	-6.49	-34.69
Ser129	-12	-17	-29
Pro156	-10.4	-15.8	-26.2
Lys132	-14	-8.93	-22.93
Trp155	-8.35	-14.4	-22.75
Asn202	-10	-12.3	-22.3
Thr154	-9.6	-9.12	-18.72
Leu157	-8.83	-4.59	-13.42
Gly193	-7.95	-4.99	-12.94
Ile99	0.12	-12.2	-12.08
Ala195	-3.05	-7.53	-10.58
Asp158	-6.3	-3.7	-10
Trp105	0.41	-9.24	-8.83
Ser153	-5.2	-1.97	-7.17
Phe211	0.32	-5.24	-4.92
Asp93	46.3	-5.32	40.98

Table S2.
Hydrogen (H)-bond persistence (%) between the NLP_{Pya} residues and different membrane GIPC molecules with different lengths (Å) between donor and acceptor atoms. Individual GIPCs are numbered according to MD simulation topology.

H-Bond	Persistence (%)	Length (Å)
GIPC 229 - ASN 194	23	2.74
GIPC 225 - ASP 104	23	2.78
GIPC 225 - ASP 104	25	2.76
GIPC 225 - ASP 104	26	2.56
GIPC 228 - ASP 205	27	2.63
GIPC 225 - ASN 196	29	2.80
GIPC 219 - GLN 213	29	2.62
GIPC 219 - GLN 213	37	2.62
GIPC 228 - ASP 205	48	2.66
GIPC 229 - ASN 194	51	2.86
GIPC 233 - ASP 205	52	2.46
GIPC 225 - HIS 128	72	2.82
GIPC 309 - HIS 128	73	2.73

Table S3.

Structural parameters for supported lipid bilayers before and after NLP_{Pya} binding. Parameter ranges as 65% confidence intervals determined from Monte Carlo resampling of the experimental data fits are provided in brackets.

	**Lipid area per molecule (Å^2)**	**Bilayer coverage (%)**	**Tail thickness (Å)**	**Head group thickness (Å)**	**Outer head group NLP_{Pya} (%)**	**NLP_{Pya} surface layer thickness (Å)**	**NLP_{Pya} surface layer coverage (%)**
POPC:sterols 7:3 + h-NLP	49.7 (48.4, 50.1)	99 (98, 100)	35.5 (34.5, 36.4)	8.1 (7.5, 8.9)	0 (0, 1)		
POPC:GIPC 6:4	65.0 (63.6, 66.8)	99 (96, 100)	26.8 (26.0, 27.4)	8.1 (7.9, 8.3)			
POPC:GIPC 6:4 + h-NLP	62.6 (61.0, 64.3)	88 (86, 90)	27.8 (27.0, 28.5)	8.8 (8.5, 9.0)	1 (0, 2)	29.0 (25.9, 31.9)	18 (17, 21)
POPC:GIPC 6:4	55.9 (54.7, 57.3)	94 (92, 96)	31.2 (30.5, 31.9)	9.1 (8.3, 10.5)			
POPC:GIPC 6:4 + d-NLP	55.5 (54.4, 56.8)	90 (88, 91)	31.5 (30.5, 31.9)	9.7 (8.3, 10.9)	0 (0, 1)	26.3 (17.0, 32.2)	10 (8, 16)
POPC:GIPC:sterols 3:4:3	68.6 (66.6, 70.0)	99 (96, 100)	25.2 (24.7, 26.0)	9.0 (8.4, 10.2)			
POPC:GIPC:sterols 3:4:3 + h-NLP	68.5 (66.0, 70.8)	96 (92, 99)	25.2 (24.5, 26.2)	10.4 (9.0, 11.7)	1 (0, 3)	34.7 (32.6, 36.5)	29 (28, 31)
POPC:GIPC:sterols 3:4:3	61.4 (60.4, 63.2)	90 (88, 93)	28.2 (27.4, 28.7)	10.0 (9.3, 11.0)			
POPC:GIPC:sterols 3:4:3 + d-NLP	61.8 (60.7, 63.0)	92 (90, 93)	28.0 (27.5, 28.5)	12.1 (11.2, 13.0)	1 (0, 4)	30.0 (27.3, 32.6)	21 (19, 25)

Movie S1.
A time-lapse recording of the binding of 300 nM NLP_{Pya} to a DOPC:GIPC:sterols 1:1:1 supported lipid bilayer in 20 mM MES, pH 5.8. Scan area: 150 nm × 150 nm; scan rate: 0.5 frame/s.

Movie S2.
A time-lapse recording of the binding of 300 nM NLP_{Pya} to a POPC:GIPC:sterols 1:6:3 supported lipid bilayer in 20 mM MES, pH 5.8. Scan area: 500 nm × 500 nm; scan rate: 0.5 frame/s.

Movie S3.
A time-lapse recording of the binding of 10 µM NLP_{Pya} to a DOPC:GIPC:sterols 1:1:1 supported lipid bilayer in 20 mM MES and 150 mM NaCl, pH 5.8. Scan area: 400 nm × 400 nm; scan rate: 0.5 frame/s.

Movie S4.
A time-lapse recording of a representative experiment showing 12 giant unilamellar vesicles filled with A594 and exposed to NLP_{Pya}-A488 in a microfluidic diffusion chamber. The movie shows the bright field image of the chamber (top) along with the red fluorescent channel for A594 (middle) and the green fluorescent channel for A488 (bottom). At 200 s, an iso-osmolar NLP_{Pya}-A488 glucose solution is introduced into the main channel and begins to diffuse into the chamber from the main channel on the left. At approximately 600 s, NLP_{Pya}-A488 reaches the vesicles and begins to bind to the membranes. The side of the membrane oriented towards the entrance of the chamber exhibits a more intense green signal compared to the opposite side of the vesicle. At approximately 850 s, the first vesicle starts to leak, as deduced from a diminishing red signal from the interior of the vesicle. The other vesicles then follow one-by-one. At 2150 s, the NLP_{Pya}-A488 solution is washed away from the main channel and subsequently also from the diffusion chamber. The NLP_{Pya}-A488 signal remains on the membrane of the vesicles.
